# Stat5 Signaling Specifies Basal versus Stress Erythropoietic Responses through Distinct Binary and Graded Dynamic Modalities

**DOI:** 10.1371/journal.pbio.1001383

**Published:** 2012-08-28

**Authors:** Ermelinda Porpiglia, Daniel Hidalgo, Miroslav Koulnis, Abraham R. Tzafriri, Merav Socolovsky

**Affiliations:** 1 Department of Pediatrics and Department of Cancer Biology, University of Massachusetts Medical School, Worcester, Massachusetts, United States of America; 2 CBSET Inc., Department of Applied Sciences, Lexington, Massachusetts, United States of America; Baylor College of Medicine, United States of America

## Abstract

Erythropoietin (Epo)-induced Stat5 phosphorylation (p-Stat5) is essential for both basal erythropoiesis and for its acceleration during hypoxic stress. A key challenge lies in understanding how Stat5 signaling elicits distinct functions during basal and stress erythropoiesis. Here we asked whether these distinct functions might be specified by the dynamic behavior of the Stat5 signal. We used flow cytometry to analyze Stat5 phosphorylation dynamics in primary erythropoietic tissue in vivo and in vitro, identifying two signaling modalities. In later (basophilic) erythroblasts, Epo stimulation triggers a low intensity but decisive, binary (digital) p-Stat5 signal. In early erythroblasts the binary signal is superseded by a high-intensity graded (analog) p-Stat5 response. We elucidated the biological functions of binary and graded Stat5 signaling using the EpoR-HM mice, which express a “knocked-in” EpoR mutant lacking cytoplasmic phosphotyrosines. Strikingly, EpoR-HM mice are restricted to the binary signaling mode, which rescues these mice from fatal perinatal anemia by promoting binary survival decisions in erythroblasts. However, the absence of the graded p-Stat5 response in the EpoR-HM mice prevents them from accelerating red cell production in response to stress, including a failure to upregulate the transferrin receptor, which we show is a novel stress target. We found that Stat5 protein levels decline with erythroblast differentiation, governing the transition from high-intensity graded signaling in early erythroblasts to low-intensity binary signaling in later erythroblasts. Thus, using exogenous Stat5, we converted later erythroblasts into high-intensity graded signal transducers capable of eliciting a downstream stress response. Unlike the Stat5 protein, EpoR expression in erythroblasts does not limit the Stat5 signaling response, a non-Michaelian paradigm with therapeutic implications in myeloproliferative disease. Our findings show how the binary and graded modalities combine to generate high-fidelity Stat5 signaling over the entire basal and stress Epo range. They suggest that dynamic behavior may encode information during STAT signal transduction.

## Abstract 2

Stat5 signaling in erythroblasts can assume either a binary, low-intensity form, essential for basal erythropoiesis, or a graded, high-intensity response, restricted to early erythroblasts and to erythropoietic stress.

## Author Summary

Hormone signaling through the erythropoietin (Epo) pathway is required both for the continuous replacement of red blood cells (RBCs) that are lost through aging (a process known as "basal erythropoiesis") and to boost tissue oxygen when bleeding, in anemia or at high altitude ("stress erythropoiesis"). A key challenge lies in understanding how extracellular Epo concentration is translated into different intracellular signals that promote transcription of proteins that are specific to basal versus stress erythropoiesis. Binding of Epo to its receptor EpoR on the surface of an erythroblast (the precursors of RBCs) triggers the addition of phosphates to a target protein Stat5; the phosphorylated Stat5 becomes activated and induces transcription. We show that the dynamic properties of the Stat5 activation signal convey additional information that specifies either basal or stress responses. During basal conditions, the Stat5 signal is low and binary in nature—an on/off switch-like response. Stress, on the other hand, triggers a distinct Stat5 response consisting of a highintensity signal that increases in a graded fashion with rising Epo concentration. We found that a mouse bearing a truncated EpoR is restricted to the low-intensity binary Stat5 signal and correspondingly fails to initiate stress erythropoiesis. Ultimately, it is the Stat5 protein level in erythroblasts that determines their ability to generate the high-intensity graded Stat5 signal in response to high Epo. These findings have therapeutic potential: targeting Stat5's high-intensity graded signal may inhibit its aberrant function in blood cell cancers without affecting its important binary response in normal cells.

## Introduction

Healthy individuals at sea level continuously generate red blood cells in a process known as “basal erythropoiesis” that is essential to life. Erythropoiesis increases by up to 10-fold its basal rate in response to hypoxic stress, as may occur at high altitude, or in response to anemia or hemorrhage. Erythropoietic rate is regulated by the hormone Erythropoietin (Epo), whose concentration in blood spans a remarkable, three orders of magnitude range, from ≈0.01 U/ml in the basal state to 10 U/ml in extreme stress. Epo exerts its effects by binding to its receptor, EpoR, a transmembrane homodimer of the cytokine receptor superfamily expressed by erythroid progenitors [1]. Epo or EpoR-null mice die at mid-gestation as a result of complete absence of mature red cells [2], and EpoR signaling is essential for both basal and stress erythropoiesis [3]–[7]. Binding and activation of the EpoR results in activation of the cytoplasmic tyrosine kinase Jak2, and in phosphorylation of EpoR cytoplasmic-domain tyrosines that act as docking sites for signaling intermediates including Stat5 [8].

A key challenge lies in understanding how EpoR signaling might differ between stress and basal conditions. This challenge is of particular relevance to clinical practice, where Epo is widely used and where erythropoietic mimetics are under intense development to maximize benefit while reducing risk [9],[10]. Here we addressed this question by studying Stat5, which, as suggested by mouse genetic models, is a key mediator of both basal and stress erythropoiesis. Thus, Stat5-null mice die perinatally due to anemia, while mice hypomorphic for Stat5 survive, but are deficient in their response to erythropoietic stress [6],[7],[11],[12].

Stat5 functions are due to two highly homologous proteins, Stat5a and Stat5b, of the Signal Transducers and Activators of Transcription (STAT) family. STAT proteins are latent cytoplasmic transcription factors that become activated by phosphorylation of a C-terminal tyrosine in response to a variety of extracellular signals [13],[14]. Stat5 is a key mediator of cell survival in erythroblasts and other hematopoietic progenitors. In addition, it is frequently constitutively active in myeloproliferative disease and in hematological malignancies [6],[7],[11],[12].

Here we asked whether the dynamic behavior of the Stat5 activation signal, namely, the way it varies with Epo concentration and with time, differs between stress and basal erythropoiesis. Previously, distinct dynamic forms of ERK or Ras signaling have been shown to specify distinct cellular responses [15],[16]. The dynamic form of a signal, however, is often masked when measured in large populations of cells whose responses are inherently variable. Analysis of a signal's dynamic properties therefore requires measurement in single cells, with relatively few such studies to date.

To address this, we analyzed Stat5 signaling using flow-cytometry, in primary murine erythroid progenitors, either in vivo or shortly following harvest (<24 h). We combined two recent flow-cytometric assays, identifying differentiation-stage-specific erythroblasts in tissue using cell-surface markers [17]–[19] and measuring their Stat5 phosphorylation signal (p-Stat5) using intracellular flow-cytometry [20]. We determined the time course and full dose-response curves of the p-Stat5 response to the entire basal and stress Epo concentration range, in freshly harvested fetal liver erythroblasts at five distinct stages of differentiation.

We found that Stat5 signals through two modalities, binary (digital) and graded (analog). We characterized these modalities using wild-type mice and an EpoR mutant mouse that we found to be restricted to the binary Stat5 signaling modality. We show that later erythroblasts generate a low intensity but decisive, binary “on” or “off” p-Stat5 signal that is both necessary and sufficient for mediating Stat5 functions in basal erythropoiesis. By contrast, in early erythroblasts Stat5 signaling is graded, reaching much higher signal intensities that are necessary for the stress response, including the upregulation of the transferrin receptor (CD71), a novel EpoR and Stat5 stress target.

The orderly transition in the modality of Stat5 signaling from early to later erythroblasts is due to decreasing Stat5 protein levels with erythroid maturation. Stat5 protein levels determine both maximal p-Stat5 signal intensity and the steepness of the Stat5 signaling response. This contrasts with EpoR expression, which does not appear to impose a limit on the maximal p-Stat5 response.

Our work shows that Stat5 signaling dynamics conveys information specifying the required functional outcome in erythroblasts. The unique combination of a steep, binary response to low Epo in the basal state, with a higher intensity graded signaling modality during stress, allows Stat5 to transduce Epo stimuli with high fidelity over its entire physiological and stress range.

## Results

### Flow Cytometric Measurement of Phosphorylated-Stat5 (p-Stat5) in Primary Erythroblasts

**Figure 1:**
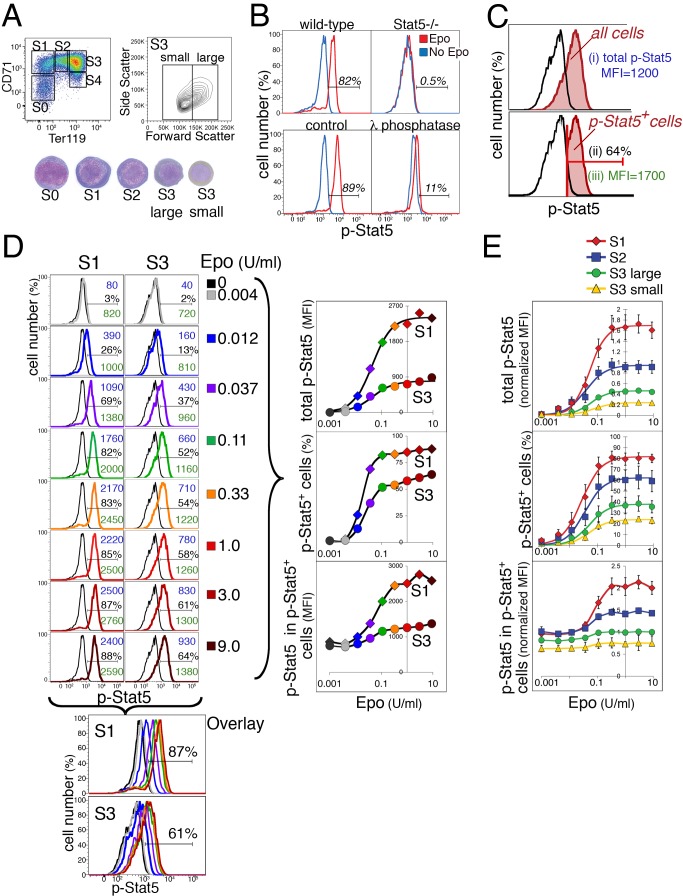
The p-Stat5 response in fetal liver. (A) Flow cytometric Ter119/CD71 profile of freshly isolated fetal liver at E13.5, fixed and permeabilized in preparation for intracellular p-Stat5 measurements. Subsets S0 to S4 (left histogram) contain erythroblasts of increasing maturation, as seen from their morphological appearance in cytospin preparations (stained with Giemsa and diaminobenzidine). The right histogram shows the further division of S3 cells into small and large subsets based on the flow-cytometric “forward scatter” parameter. (B) Specificity of the Alexa 647-conjugated anti-p-Stat5 antibody (BD Biosciences # 612599). Upper panels, response of S1 cells from either wild-type or Stat5^−/−^ fetal livers to Epo stimulation (2 U/ml) for 15 min (red histograms). Blue histograms are baseline fluorescence in the absence of Epo. Lower panels, Epo-stimulated (2 U/ml; 15 min, red histograms) or unstimulated (blue histograms) S1 cells in wild-type fetal liver, either treated or untreated with λ-phosphatase prior to p-Stat5 staining. Numbers in all panels indicate the fraction (%) of p-Stat5 positive cells within the indicated horizontal gates. (C) The three measures used to analyze the p-Stat5 response to Epo (9 U/ml, 15 min, red histogram) in S3 erythroblasts. The black histogram corresponds to pre-stimulation cells of the same subset. (i) *“total p-Stat5 median fluorescence intensity (MFI)”* (upper panel), the p-Stat5 MFI of the entire S3-subset population, represented by the shaded red histogram. This measure does not distinguish between signaling and nonsignaling cells. (ii) *“p-Stat5^+^ cells(%)”* (lower panel), cells within the p-Stat5^+^ gate, shaded in red, expressed as a fraction (percent) of all cells in the Epo-stimulated S3 subset. This is an estimate of the number of signaling cells. The placement of the p-Stat5^+^ gate was determined by reference to the baseline, pre-stimulation histogram (in black), so that no more than 1% of the unstimulated population is included within the p-Stat5^+^ gate. (iii) *“p-Stat5 in p-Stat5^+^ cells”* (lower panel), estimates the p-Stat5 MFI in signaling cells only. (D) Response of S1 and S3 erythroblasts to Epo stimulation. Freshly isolated fetal liver cells were deprived of Epo for 90 min and were then stimulated with a range of Epo concentrations as indicated, from 0.004 to 9 U/ml, for 15 min. Colored flow-cytometry histograms correspond to Epo-stimulated cells, black histograms in each panel correspond to unstimulated cells (Epo = 0). An overlay of the responses is shown in the two lowest panels. For each Epo concentration, three measures of the p-Stat5 response, as illustrated in (C), are noted next to each flow-cytometry histogram in blue, black, and green, corresponding to the total p-Stat5 MFI, to the p-Stat5^+^ cells (%), and to the p-Stat5 in p-Stat5^+^ cells, respectively. Each of these measures is then plotted as a function of Epo concentration (right panels); the color of each symbol in these plots corresponds to the color of the respective flow-cytometry histogram for the same Epo concentration. (E) The p-Stat5 response to a range of Epo concentrations at 15 min post-stimulation. Summary of five independent experiments similar to Panel D. Data (mean ± SE) for each experiment were normalized by expressing each p-Stat5 MFI reading as a ratio to the p-Stat5 MFI in p-Stat5^+^ cells of the “S3 large” subset following stimulation with 1 U/ml Epo for 15 min. Data in the upper two panels were fitted with Hill curves.

Murine erythropoiesis takes place in fetal liver between embryonic days 12 (E12) and 15. To examine intracellular Stat5 activation by phosphorylation, we fixed and permeabilized fresh fetal liver cells, which we then labeled with an AlexaFluor647-conjugated antibody specific to the Stat5 C-terminal phosphotyrosine. In addition, we labeled the cells' surface with antibodies directed at CD71 and Ter119, which may be used to stage erythroblast maturation [17],[18],[21],[22]. We distinguished subsets S0 to S4 in the fixed fetal liver, with the earliest erythroid cells in S0, maturing into increasingly differentiated erythroblasts in S1 through S4; S3 is further subdivided into earlier, large cells and more differentiated, small cells (Figure 1A). Unless otherwise stated, “S3” below refers to “S3 large.” All cells in subsets S1 to S3 are Epo-dependent erythroid precursors; S0 is composed of earlier, Epo-independent erythroid progenitors (70%) and non-erythroid cells (30%, [18]). Following stimulation of freshly isolated fetal liver cells with Epo, we measured an Epo-dependent signal with the anti-phosphorylated-Stat5 (p-Stat5) antibody (Figure 1B). This signal was specific to the active, p-Stat5, since it was obtained in wild-type, but not in Stat5^−/−^ fetal liver (Figure 1B, upper panels). Further, the p-Stat5 signal was lost if, following Epo stimulation, fixed cells were incubated with λ phosphatase (Figure 1B, lower panels). Work below also confirmed, with the use of a Stat5 mutant, that the signal is specific to the C-terminal Y694 residue.

### Erythroid Maturation Determines the p-Stat5 Response

We stimulated freshly isolated fetal liver cells with Epo and examined the resulting p-Stat5 response in each of the fetal liver subsets (Figure S1A; Figure 1C–E). We measured three aspects of the p-Stat5 fluorescence signal (Figure 1C). First, “total p-Stat5” corresponds to the p-Stat5 median fluorescence intensity (MFI) of the entire subset population; the total p-Stat5 MFI of all S3 subset cells in the red histogram, upper panel of Figure 1C, is 1,200 fluorescence units. This measure includes both signaling and non-signaling cells. Second, we measured the fraction of cells that are “p-Stat5 positive” (p-Stat5^+^), lying within the p-Stat5^+^ gate (Figure 1C, lower panel), as an estimate of the fraction of signaling cells. The placement of the p-Stat5^+^ gate was determined by reference to the baseline, pre-stimulation histogram (black histogram, Figure 1C, lower panel), which was closely similar to that of cells stained with an isotype-control antibody in place of the anti-p-Stat5 antibody. Last, we measured the “p-Stat5 in p-Stat5^+^ cells,” which estimates the p-Stat5 MFI in signaling cells only (Figure 1C, lower panel, where p-Stat5 in p-Stat5^+^ cells is 1,700 fluorescence units).

Using these measures, we examined the p-Stat5 response to Epo at 15 min post-stimulation, when a peak response is attained (see time course of activation, Figure S1B). The p-Stat5 signal intensity was highest in S1, decreasing with erythroid maturation through subsets S2 and S3 (Figure S1A). In the earliest, S0 subset, only ∼25% of cells responded to Epo, suggesting that the p-Stat5 response pathway becomes fully activated only with the onset of Epo dependence at the transition from S0 to S1, when a number of key transcriptional and epigenetic changes take place in erythroid progenitors (Figure S1A,C [18],[19]).

We contrasted the response of S1 and S3 cells to a range of Epo concentrations encountered in physiological (<0.05 U/ml) and hypoxic-stress conditions (0.05 to 10 U/ml; Figure 1D; each histogram in the left panels is represented as a data-point of the same color in the dose/response curves in the right panels). S1 cells generated a graded increase in total p-Stat5 in response to increasing Epo (Figure 1D, right upper panel), which reflected a graded increase in both the number of signaling cells (p-Stat5^+^ cells, Figure 1D, right middle panel) and in the signal intensity of signaling cells (Figure 1D, lower right panel). “S3 large” ( = “S3”) cells attained a ≈4-fold lower signal than S1 cells. The S3 cell population also showed a graded increase in total p-Stat5 with increasing Epo stimulation (Figure 1D, right upper panel). However, this was principally the result of an increase in the number of signaling cells with Epo concentrations (Figure 1D, right middle panel); the p-Stat5 signal intensity within signaling cells remained relatively constant (Figure 1D, right lower panel). A summary of five independent experiments for all erythroid subsets shows that these dose/response characteristics are reproducible (Figure 1E).

### Graded versus Binary Signaling in Cell Populations

**Figure 2:**
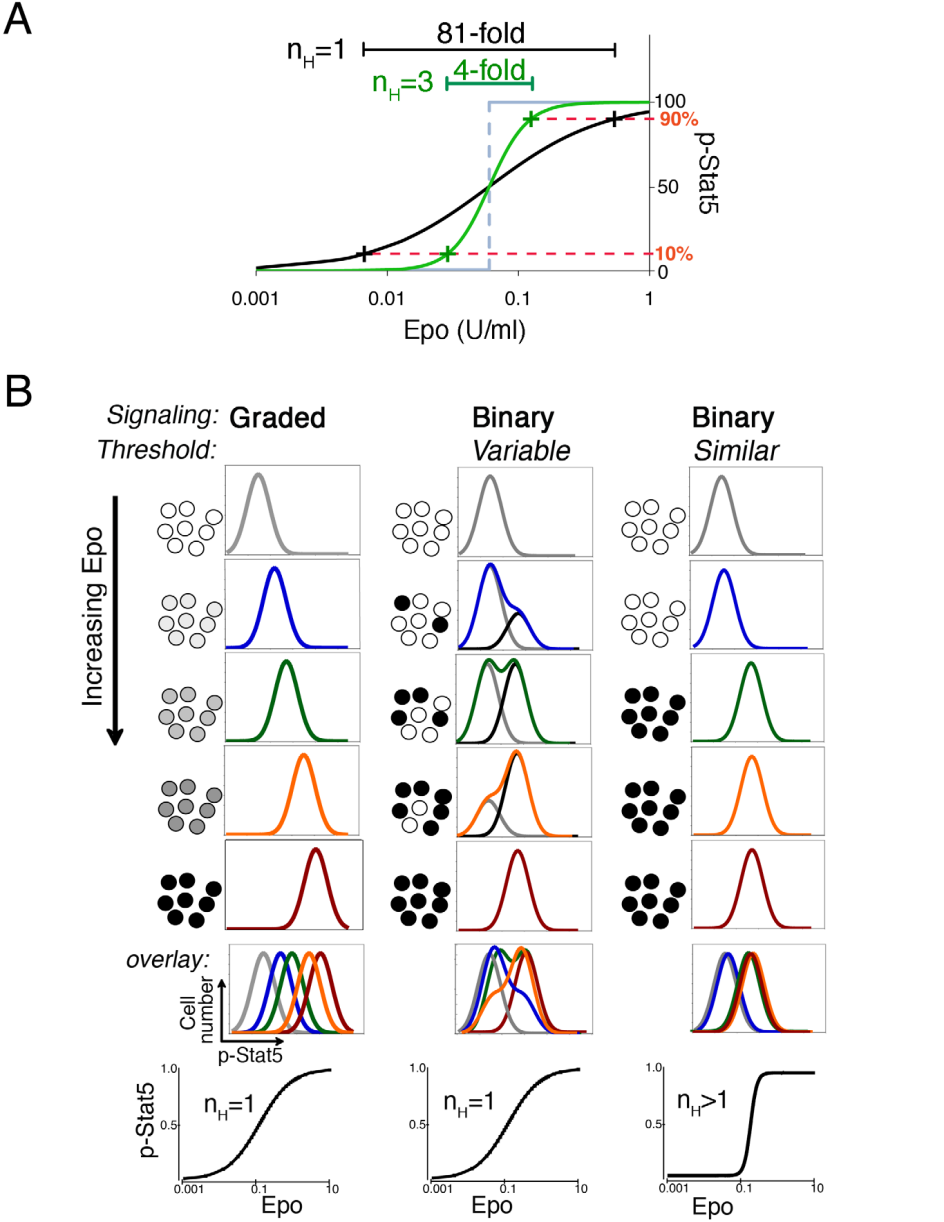
Measurement of binary and graded signaling responses by flow cytometry. (A) Dose/response curves for graded and binary (switch-like) responses. The grey line shows an idealized switch response. An infinitesimally small change in stimulus such as Epo results in an increase of response from 0% to 100%. The black curve shows a graded response with n_H_ = 1. An 81-fold increase in stimulus is required to shift the response from 10% to 90% of maximum. The green curve shows a steep, switch-like or binary dose/response curve (n_H_ = 3). Only a 4-fold change in stimulus is required in order to generate a similar increase in response. (B) Contrasting graded and binary signaling responses: three hypothetical examples. In a graded signaling response (left panels), increasing Epo concentration results in a graded increase in p-Stat5 in individual cells, represented by increasingly darker shades of grey. Simulations of the corresponding flow-cytometric profiles show that increasing Epo concentration causes a gradual shift of the p-Stat5 fluorescence histogram to the right. A plot of the total p-Stat5 median fluorescence intensity (MFI) versus Epo concentration has Michaelian kinetics with a Hill coefficient (n_H_) of 1 (lower left panel, please note a log scale was used for the *x*-axis). In binary signaling (middle and right panels), the p-Stat5 signal in individual cells can only assume two states, either “off” (white) or “on” (black), but intermediate states (shades of grey) are unstable. Two distinct cases of binary signaling are illustrated that differ in their threshold responses. In “variable threshold” (middle panels), the threshold at which Epo causes a cell to switch from “off” to “on” varies substantially between cells of the population. Consequently, increasing Epo concentration causes a gradual increase in the number of cells that are p-Stat5^+^ (“on”). The simulated flow-cytometric histograms at each Epo concentration (in color) are each the sum of two histograms, corresponding to cells that are “off” (light grey histograms) and cells that are “on” (black histograms). The median fluorescence of the “on” and “off” histograms each remain unaffected by Epo concentration, but as Epo increases, the number of cells in the “on” histogram, reflected by its height, increases, with a corresponding decrease in the height of the “off” histogram. Although individual cells have binary responses, there is a graded increase in the MFI of the colored histograms representing the whole population. Therefore, a plot of total p-Stat5 MFI versus Epo concentration shows a graded response (here, the Hill coefficient is n_H_ = 1). In the case of cells with binary responses and similar threshold (right panels), cells switch from “off” to “on” within a much narrower Epo concentration range. Consequently, the response of the whole population reflects the response of individual cells more closely. Flow-cytometric histograms representing the population tend to be in one of two principal positions, corresponding to the “on” or to the “off” states. A plot of total p-Stat5 MFI versus Epo concentration is steep, reflected by a high Hill coefficient (n_H_>1).

In spite of wide variation in the number of responding cells, the p-Stat5 signal intensity of S3 cells with a positive p-Stat5 response (p-Stat5 MFI in p-Stat5^+^ cells) remained relatively constant (Figure 1E, middle and lower panels). We found that maturation-stage equivalent cells in adult tissue behaved similarly (“EryA” erythroblasts, Figure S2). Together, these findings raised the possibility that S3 cells may be generating a binary p-Stat5 response. Under this hypothesis, p-Stat5 activation in individual S3 cells would be switch-like, with cells either expressing their maximal p-Stat5 levels regardless of Epo concentration (and are “on”) or failing to respond and remaining “off.” In an idealized switch-like response, an infinitesimally small increase in stimulus in the region of the stimulus threshold can cause the response to increase from 0% to 100%. The Hill coefficient of this idealized step-like dose/response curve approaches infinity (Figure 2A, gray step response). Switch-like responses in biology, however, are only approximations of this idealized case, in that the switch in response from 0% to 100% requires a small but finite increase in the stimulus. Goldbeter and Koshland called switch-like biological responses “ultrasensitive” and defined them as steeper than the graded hyperbolic Michaelis-Menten curve—that is, responses with a Hill coefficient (n_H_) larger than 1 [23]. In a graded response (n_H_ = 1), the stimulus needs to increase 81-fold in order to increase the response form 10% to 90% of maximum. By contrast, in a more switch-like, steeper response, with n_H_ = 3, only a 4-fold increase in stimulus is required for a similar change in response (Figure 2A). Examples of effective switch-like responses include the cooperative binding of oxygen to hemoglobin (n_H_ = 2.7) and the MAPK pathway in *Xenopus* oocytes (n_H_ = 4 or 5) [24],[25].

A binary p-Stat5 response in single cells may sometimes appear to be graded when the signal is measured in a population of cells. This is illustrated in Figure 2B, which contrasts three hypothetical cases of signaling cells. In the first case (Figure 2B, left panels), there is a graded increase in signal within individual cells in response to increasing Epo concentration, resulting in a graded increase in the total p-Stat5 MFI at the population level. The corresponding Epo dose/p-Stat5 response curve has a Hill coefficient of 1 (Figure 2B, left lower panel). The two hypothetical cases of binary signaling (Figure 2B, middle and right panels) differ from each other only in the Epo threshold at which cells respond with a p-Stat5 signal. In the “variable threshold” example, individual cells vary substantially with respect to the Epo concentration at which they switch from “off” to “on.” The measured p-Stat5 signal, which is the sum of the signals generated by a large population of cells, increases in a graded manner with increasing Epo concentration (n_H_ = 1) (Figure 2B, lower middle panel). The flow-cytometry histograms for each Epo dose (in color) are a composite of two underlying histograms, of non-signaling cells (in grey) and signaling cells (in black). Only the amplitudes of the black and grey histograms change with Epo concentration, while their MFI remains constant. However, the MFI of the composite, color histogram increases in a graded manner with Epo dose. By contrast, in the second binary signaling example (Figure 2B, right panels), cells have a similar threshold to Epo stimulation, so that the entire cell population switches from “off” to “on” within a narrow Epo concentration range. This results in the population response resembling the binary responses of individual cells, with a much steeper Epo dose/p-Stat5 response curve that is characterized by a high Hill coefficient (n_H_>1) (Figure 2B, lower right panel).

A graded p-Stat5 response in the S3 population (Figure 1E, top panel) does not therefore preclude the possibility that individual S3 cells have binary responses that are masked by a variable threshold to Epo (as in Figure 2B, middle panel).

### A Binary Low-Intensity p-Stat5 Signal in EpoR-HM Erythroblasts

**Figure 3:**
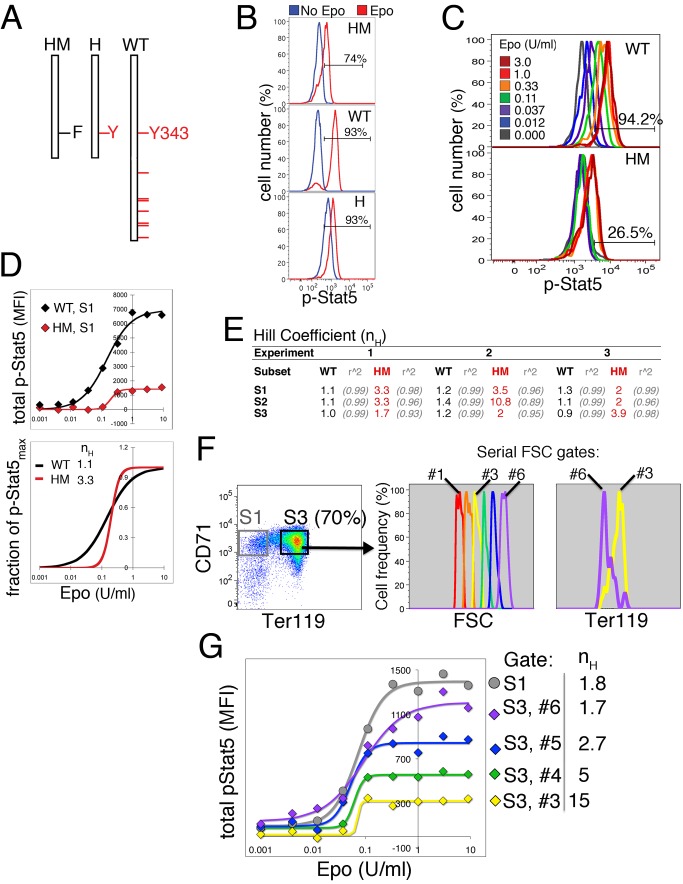
Binary p-Stat5 signaling in EpoR-HM mice and in mature wild-type S3. (A) Representation of the cytoplasmic domains of wild-type EpoR or its truncated mutants, EpoR-H and EpoR-HM. Tyrosine residues are represented by red lines. Tyrosine 343 is the only remaining tyrosine in EpoR-H, and is mutated in EpoR-HM. (B) The p-Stat5 response to Epo (2 U/ml, 30 min) in S1 cells from wild-type (WT), EpoR-H (H), or EpoR-HM (HM) fetal livers on E13.5. Percentage of cells in the p-Stat5^+^ gate is indicated. (C) Fluorescence p-Stat5 histogram overlay of S1 cells from E13.5 wild-type (top) or EpoR-HM (bottom), stimulated with a range of Epo concentrations for 30 min. Representative of three similar experiments. S1 cells in each case are pooled from several fetal livers of the same genotype. (D) Epo dose/p-Stat5 response curves, in wild-type or EpoR-HM S1 cells; the p-Stat5 MFI data correspond to the histograms shown in (C). Data are fitted with Hill curves. The Hill curves that were fitted to the data in the top panel were re-plotted in the bottom panel and are shown as a fraction of the maximal p-Stat5 response (p-Stat5_max_). The p-Stat5_max_ was calculated from fitting the Hill equation to the experimental “total p-Stat5” data. Hill coefficients (n_H_) are indicated. (E) Lower panel, table summarizing the Hill coefficients (n_H_) obtained by fitting the Hill curve to plots of “total p-Stat5 versus Epo concentration,” for each of subsets S1 to S3, in each of three independent experiments. Fetal liver cells were pooled from three or four embryos of each genotype in each experiment. Differences between n_H_ for EpoR-HM and WT are significant at *p* = 0.01, paired *t* test. *R* ^2^ is Pearson's product moment correlation coefficient, correlating experimental data with values predicted by the Hill equation for the corresponding Epo concentrations. (F) CD71/Ter119 profile of E14.5 fetal livers (left panel). The S3 subset was further divided into serial FSC gates, each corresponding to 300 channels (middle panel). The right panel shows an overlay of Ter119 expression in FSC gates #3 and #6. (G) Epo dose/p-Stat5 response curves for the S3 FSC gates #3–6 and for S1 cells in the same fetal liver. Gate number and corresponding Hill coefficient are shown for each curve.

We studied p-Stat5 signaling in the EpoR-H and EpoR-HM mouse strains, in which the respective EpoR truncation mutants are “knocked-in” at the wild-type EpoR locus, replacing wild-type EpoR (Figure 3A, [3]). EpoR-H lacks seven of the eight cytoplasmic domain tyrosines. EpoR-HM is similarly truncated but in addition contains the Y343F mutation and therefore lacks tyrosine docking sites for Stat5.

S1 cells from EpoR-H fetal livers generated a p-Stat5 signal equivalent to that of wild-type cells, but had a high p-Stat5 background in the absence of Epo stimulation, consistent with a previously identified negative regulatory function for the EpoR carboxy-terminal domain (Figure 3B, lower panel; [26]). S1 cells from EpoR-HM fetal liver, by contrast, generated only a low-intensity p-Stat5 response to Epo, consistent with previous studies (Figure 3B, upper panel; [27]). A full Epo dose/p-Stat5 response analysis revealed that the maximal p-Stat5 signal generated by S1 cells in EpoR-HM was ≈3–4-fold lower than in wild-type S1, resembling in intensity p-Stat5 signals generated by more mature, wild-type S3 cells (Figure 3C–D, Figure S3A). Strikingly, in addition to their lower p-Stat5 intensity, the EpoR-HM S1 response was binary (Figure 3C–E), resembling the hypothetical example of binary signaling in a population of cells with similar Epo thresholds (Figure 2B, right panels). Thus, unlike wild-type S1, the p-Stat5 fluorescence histograms in EpoR-HM S1 are in one of two clusters, either “off” or “on” (Figure 3C, lower panel). The switch from “off” to “on” occurs at ∼0.3 U/ml (see apparent K_m_ values for the EpoR-HM dose/response curve, Figure S3B). This binary behavior was reflected in the steep Epo dose/p-Sta5 response curve for EpoR-HM S1 cells (Figure 3D). In each of three independent experiments, the Hill coefficients found for each of the EpoR-HM fetal liver subsets were consistently higher than in wild-type littermate controls (Figure 3E), with n_H_ for S1 cells ranging between 2 and 3.5. Taken together, S1 cells in EpoR-HM have lost the high-intensity graded signaling mode characteristic of this subset. The residual signal is of low intensity, similar to that of S3 cells, and is binary in nature.

### Binary Signaling in S3 Cells of Similar Maturation Stage

The S3 subset consists of a spectrum of erythroblast maturational stages with varying size and hemoglobin expression (Figure 1A; [18]). Since variability between cells may mask binary signaling properties, we attempted to subdivide S3 cells into more uniform subsets. Maturation is associated with a decrease in cell size. We made use of this trend to digitally sub-divide the S3 population of cells in an E14.5 fetal liver into a series of smaller subsets, based on their forward scatter (FSC) parameter, which is a function of cell size (Figure 3F). We confirmed that increasingly smaller cells were indeed increasingly mature by comparing Ter119 expression in each of the FSC gates. As expected, larger cells in FSC gate #6 expressed less Ter119 than smaller cells in FSC gate #3 (Figure 3F). We proceeded to analyze the Epo dose/p-Stat5 response properties of cells in individual FSC gates, and found that signaling by smaller and more mature cells was binary, with high Hill coefficients; the steepness of the dose/response curve decreased progressively in less mature cells, while the p-Stat5 intensity increased. For comparison, the dose/response curve for the S1 subset was much less steep (n_H_ = 1.8), similar to that found for the least mature cells within S3 (Figure 3G).

This analysis suggests that the most mature cells within S3 generate the lowest p-Stat5 signal intensity, and have the steepest dose/response curves, giving rise to an overall binary response pattern. We carried out a similar analysis on S3 cells from a younger, E12.5 embryo, in which the most mature cells within S3 had not yet developed (Figure S4). There were fewer cells in the low FSC gates of the E12.5 embryo, and these were less mature than in corresponding gates of the E14.5 embryos, as indicated by Ter119 expression (Figure S4A, right panels). All dose/response curves in the E12.5 embryos had lower Hill coefficients (n_H_∼1.2 to 1.8) and hence a more graded response (Figure S4B). Of interest, cells in FSC gate #3 in the E12.5 fetal liver generated a similar p-Stat5_max_ signal intensity to that of cells in FSC gate #4 of the E14.5 fetal liver. However, the steepness of the dose/response curve of the two cell types was markedly different (n_H_ = 1.6 and 5, respectively) (Figure S4C), in line with their differing maturational state. This analysis suggests that, for a given maximal p-Stat5 signal intensity, more mature cells generate a steeper dose/response curve.

### SOCS3 Expression Increases with Erythroblast Maturation, Modulating the p-Stat5 Response

We investigated factors that might account for the gradual decrease in the p-Stat5 response as cells mature (Figure 1E). Differentiation of S1 into “S3 small” cells takes 24 to 48 h and entails large changes in gene expression [18]. We examined the potential role of two established Jak2 and Stat5 negative regulators, Shp1 (Figure S5) and SOCS3 (Figure S6) [28]. Shp1 mRNA expression decreases with maturation from S0 to S3 (Figure S5A). There was no significant difference in either the time course of the p-Stat5 response to Epo or in the dose/response curve, between Shp1^−/−^ (C57BL/6J-Ptpn6^me^/J) fetal liver and littermate controls (Figure S5B–C). In contrast to Shp1, SOCS3 mRNA expression increased with the transition from S1 to S3 (Figure S6A). Knock-down of SOCS3 expression successfully prevented its induction following Epo stimulation (Figure S6B). In S1 cells, SOCS3 knock-down had no effect on the initial p-Stat5 response, but it prevented the decline in p-Stat5 that was invariably detected by 2 h post-stimulation (Figure S6C, left panels). This pattern is consistent with the known negative feedback role of SOCS3 in Stat5 signaling [29],[30]. In contrast to S1, knock-down of SOCS3 in S3 cells increased the peak p-Stat5 signal intensity at 15 min, suggesting that the lower p-Stat5 signal intensity in S3 is in part the result of their higher SOCS3 expression (Figure S6C, right panels).

### A Decrease in Stat5 Protein Levels with Erythroid Maturation Closely Correlates with a Decreasing p-Stat5 Response

**Figure 4:**
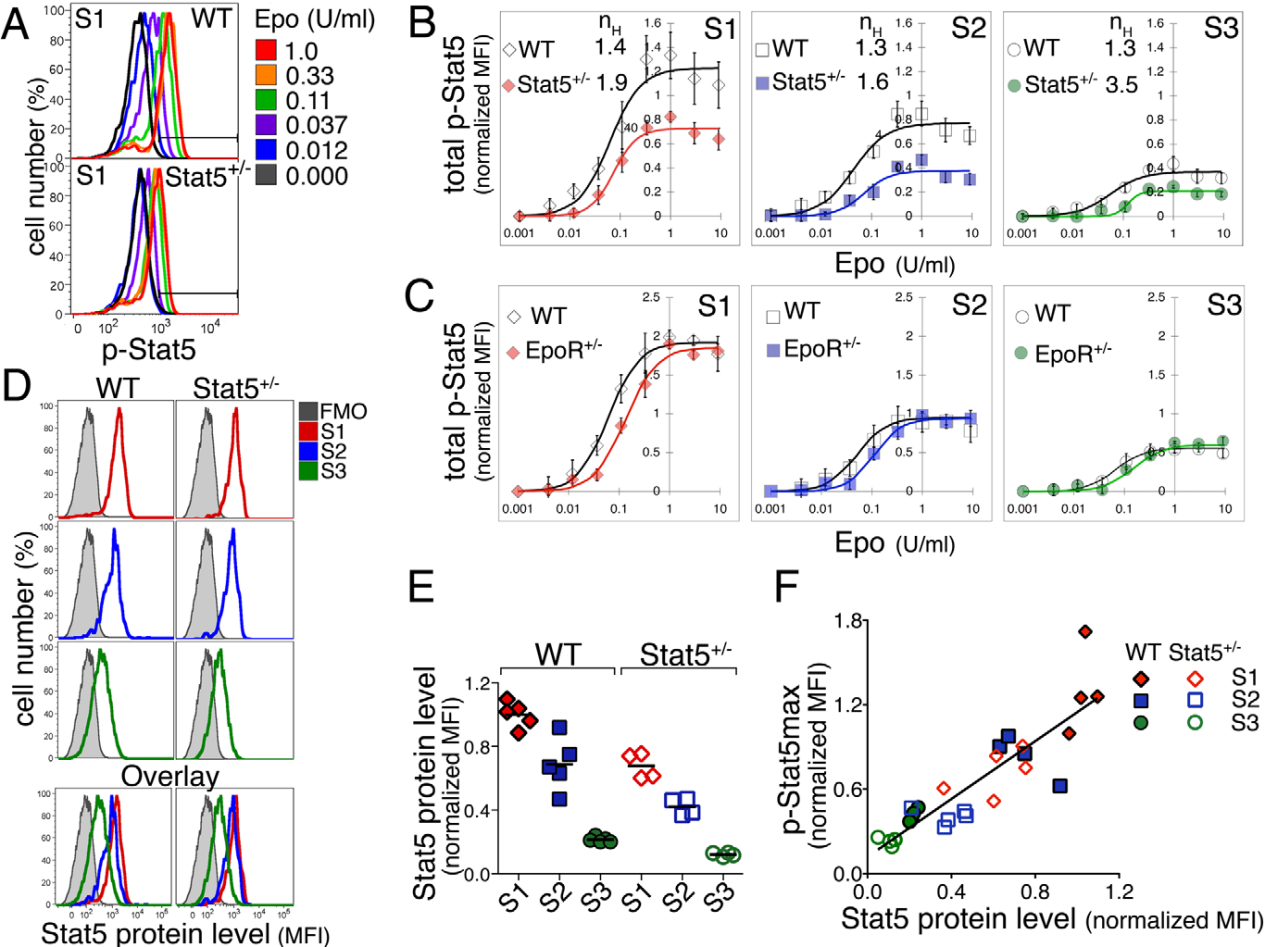
Maximal p-Stat5 signal intensity (p-Stat5_max_) is linearly correlated with Stat5 protein levels. (A) The p-Stat5 response of S1 cells from Stat5^+/−^ fetal liver and from littermate wild-type controls. Representative p-Stat5 fluorescence histograms are shown for the indicated Epo concentrations. (B) Plots of “total p-Stat5 versus Epo concentration” in Stat5^+/−^ and in wild-type littermate fetal livers in experiments similar to (A), fitted with Hill curves. Data (mean ± SE) from *n* = 7 Stat5^+/−^ embryos and 6 littermate controls, each analyzed separately. p-Stat5 MFI was normalized as in Figure 1E. Parameter values used for fitting the Hill curves and goodness of fit information are in Figure S7A. (C) Plots of “total p-Stat5 versus Epo concentration” in EpoR^+/−^ and in wild-type littermate fetal livers, fitted with Hill curves. Data (mean ± SE) from *n* = 4 EpoR^+/−^ embryos and 3 littermate controls, each analyzed separately. p-Stat5 MFI was normalized as in Figure 1E. Parameter values used for fitting the Hill curves and goodness of fit information are in Figure S7C. (D) Stat5 protein levels in S1 to S3, in representative wild-type (WT) and Stat5^+/−^ embryos, using flow-cytometric measurements with an anti-Stat5 antibody, characterized in Figure S7D using Stat5^−/−^ embryos. FMO, “fluorescence minus one” control, in which cells were labeled for all parameters (Ter119, CD71, live/dead stain) except one: the anti-Stat5 antibody was omitted and replaced with an isotype-control antibody. (E) Stat5 protein levels in subsets S1 to S3 in wild-type or Stat5^+/−^ embryos. Individual data points correspond to data from individual embryos, measured as in panel D. Stat5 protein is expressed as a ratio to the average fluorescence signal for S1 cells in all wild-type embryos. (F) Linear correlation between Stat5 protein levels and p-Stat5_max_, across all subsets in Stat5^+/−^ and wild-type embryos (*R* ^2^ = 0.85). Data points correspond to individual embryos. Stat5 protein levels are as in (D). p-Stat5_max_ was determined by fitting Hill curves to individual embryo “total p-Stat5 versus Epo concentration” analyses, with p-Stat5 normalized as in Figure 1E.

We examined the potential role of changes in EpoR or Stat5 protein levels during erythroblast maturation. To this end we investigated embryos heterozygous for the null allele of either Stat5 or EpoR (Figure 4). An Epo dose/p-Stat5 response analysis in fetal liver cells from Stat5^+^/^−^ embryos showed a clear decrease in the p-Stat5 signal across the entire Epo concentration range in all fetal liver subsets S1 to S3, compared with wild-type controls (see representative example in Figure 4A; a dataset of 7 Stat5^+^/^−^ and 6 wild-type littermates embryos is summarized in Figure 4B). Fitting Hill curves to the dose/response data yielded three parameters: the apparent K_m_, the maximal p-Stat5 signal at high Epo concentrations, defined as “p-Stat5_max_,” and the Hill coefficient, n_H_ (Figure S7A). In addition to the clear decrease in p-Stat5_max_ in all subsets of the Stat5^+^/^−^ fetal liver (Figure 4A,B, Figure S7A), the p-Stat5 response curve was steeper, reflected in a higher Hill coefficient (Figures 4B and S7A). There was also a shift to the right (increase in the apparent K_m_) in Stat5^+^/^−^ S3 cells. The apparent K_m_ reflects a number of separate sequential interactions: binding of Epo to the EpoR, Jak2 activation, Jak2 phosphorylation of the EpoR, binding of Stat5 to the phosphorylated EpoR, and phosphorylation of Stat5. A change in the apparent K_m_ can in principle be due to alterations anywhere in this pathway. Reduced expression of Stat5 in Stat5^+^/^−^ embryos may affect recruitment of Stat5 to EpoR phosphotyrosines, potentially explaining the higher apparent K_m_.

To assess the relation between Stat5 protein levels and the maximal p-Stat5 response more precisely, we measured Stat5 protein levels in individual cells within each of the Stat5^+/−^ and wild-type embryos, using anti-Stat5 antibodies and flow cytometry, a method that we verified using the Stat5-null fetal livers (Figure S7D). Stat5 protein levels in wild-type fetal liver decreased with maturation, being highest in S1 and 4-fold lower in “S3 large” cells (Figure 4D,E, closed symbols). A similar pattern was observed in Stat5^+/−^ embryos, but for each corresponding subset, Stat5 protein levels were approximately halved compared with wild-type cells (Figure 4D,E, open symbols). There was a linear correlation (*R* ^2^ = 0.85) between Stat5 protein levels in each of the wild-type or Stat5^+/−^ fetal liver subsets and their corresponding maximal p-Stat5 response (p-Stat5_max_, Figure 4F; p-Stat5_max_ was determined by fitting a Hill curve to data from each embryo). These findings suggest that decreased Stat5 protein levels may cause the decrease in the p-Stat5 response with cell maturation in wild-type embryos (Figure 1E), as well as the reduced p-Stat5_max_ in Stat5^+/−^ embryos (Figure 4A,B).

### The p-Stat5 Response in the EpoR^+/−^ Fetal Liver

We examined the Epo dose/p-Stat5 response in fetal livers derived from EpoR^+/−^ embryos and their littermate controls (Figure 4C). EpoR^+/−^ fetal livers had an approximately 2-fold decrease in EpoR mRNA (Figure S7B). Unlike the Stat5^+/−^ embryos, there was no change in p-Stat5_max_ in EpoR^+/−^ fetal liver. Instead, the EpoR^+/−^ dose/response curves were shifted to the right, with a 2-fold increase in the apparent K_m_ (Figure 4C, Figure S7C), raising the possibility that a doubling in Epo concentration compensated for the reduced expression of EpoR. Therefore, although EpoR^+/−^ fetal liver requires a higher Epo concentration to elicit a given p-Stat5 signal, the likely reduced cell-surface EpoR in these embryos appears not to limit the maximal p-Stat5 response.

To investigate this further, we asked whether EpoR^+/−^ fetal livers in fact have less EpoR available for activation. We sorted Ter119 negative cells, equivalent to subsets S0 and S1, from E13.5 fetal livers of either wild-type or EpoR^+/−^ embryos. We briefly stimulated the cells with a high Epo concentration that would be expected to generate a maximal p-Stat5 response (2 U/ml for 5 min). We used quantitative Western blot analysis to examine both p-Stat5 and phosphorylated EpoR (p-EpoR) in each fetal liver (Figure S8). This analysis showed that EpoR^+/−^ fetal liver cells had reduced p-EpoR but not reduced p-Stat5; specifically, the ratio of p-EpoR to p-Stat5 in each fetal liver was significantly higher in wild-type compared with the EpoR^+/−^ embryos (1.7±0.02 versus 1.1±0.08, *p* = 0.002; Figure S8B). These results support the conclusion that EpoR expression in primary fetal liver cells is present at sufficiently high levels so as not to limit the maximal p-Stat5 signal.

### Exogenous Stat5 Protein Endows EpoR-HM and Wild-Type S3 with a Graded, High-Intensity p-Stat5 Response

**Figure 5:**
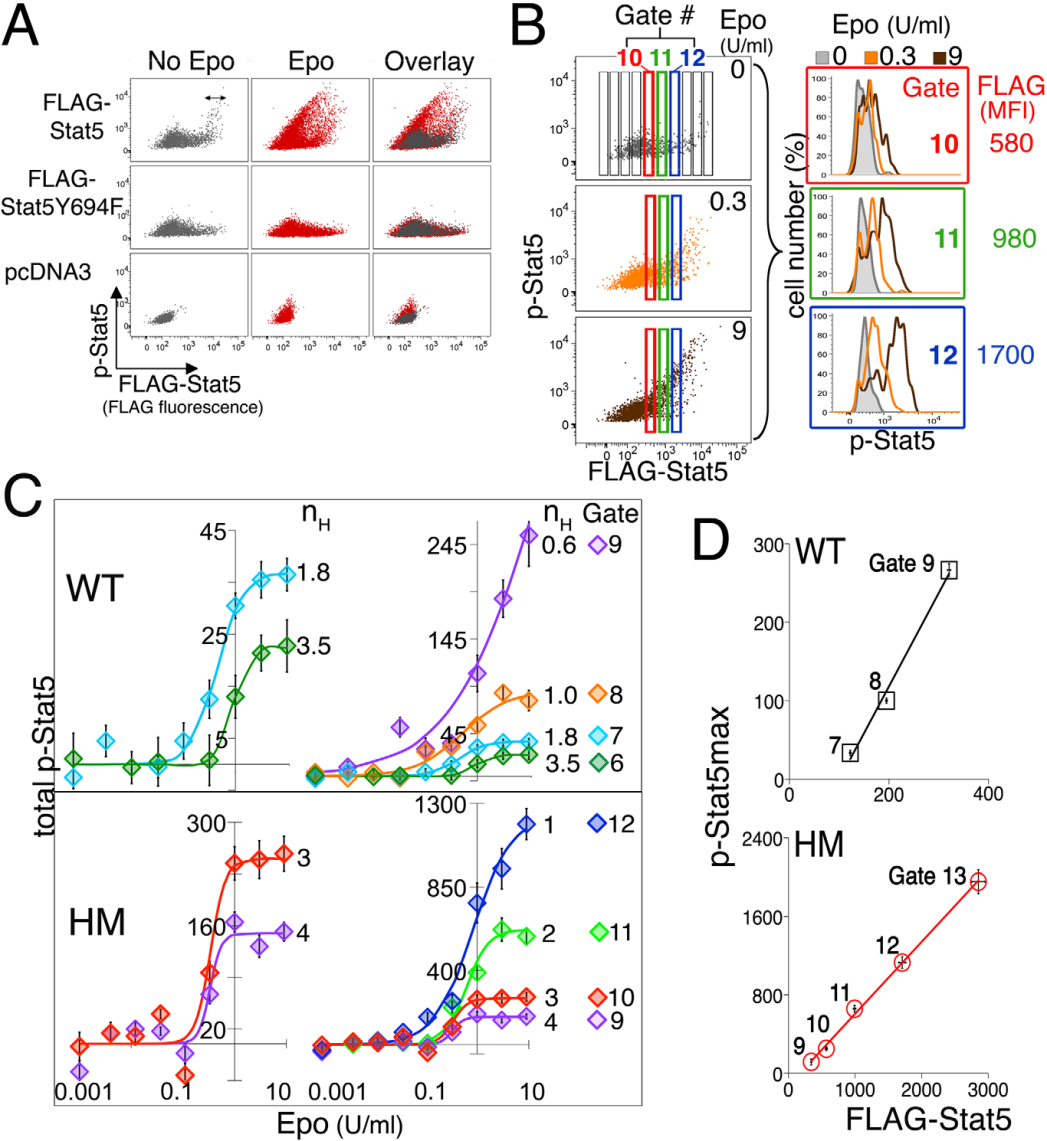
High exogenous Stat5 levels rescue high-intensity graded p-Stat5 signaling in wild-type and EpoR-HM S3 cells. (A) The p-Stat5 response to Epo in S3 cells expressing exogenous FLAG-Stat5. Wild-type fetal liver cells were electroporated with either FLAG-tagged Stat5a ( = FLAG-Stat5, C-terminal tag, top panels), FLAG-tagged STAT5Y694F (middle panels), or “empty vector” (pcDNA3, lower panels). Following overnight incubation in Epo (0.2 U/ml), cells were deprived of Epo for 3 h, and then either left unstimulated (left panels) or were stimulated with Epo for 30 min (response to Epo = 9 U/ml is shown; response to Epo concentrations 0.04 to 9 U/ml are shown in panels B, C). Double-headed arrows indicate persisting p-Stat5 following a 3h Epo deprivation, seen only in cells expressing high levels of FLAG-Stat5. (B) Analysis strategy for the experiment described in panel A. Dot plots of the p-Stat5 response versus FLAG-Stat5 levels are shown for three of the nine Epo concentrations examined (left panels; Epo = 0, 0.33, or 9 U/ml) in EpoR-HM S3 erythroblasts. Each dot plot was subdivided into narrow vertical gates, each containing cells of similar FLAG-Stat5 levels. Three of these gates, numbered 10 to 12, are highlighted in red, green, and blue, respectively. p-Stat5 responses to the three Epo concentrations in a given gate (left panels) were overlayed in a single histogram (right panels). For example, responses of cells in gate 10 (red) in each of the three left panels are all overlayed in the top right panel (framed red). (C) The “total p-Stat5” response to each of nine Epo concentrations for each vertical gate in panel B, fitted with a Hill curve. Analysis is shown for both wild-type (WT) S3 cells and for EpoR-HM S3 cells (HM). Gate numbers and Hill coefficients are indicated next to each curve. Data points are “total p-Stat5” (MFI±sem) for cells in a given gate. Each of the “total p-Stat5” MFI measurements was corrected by subtracting background fluorescence, given by the “total p-Stat5” MFI of cells expressing FLAG-Stat5Y694F in the same gate (see panel A). Gates 6 and 7 for wild-type cells and gates 9 and 10 for HM cells are re-plotted with an expanded *y*-axis. (D) Linear correlation between FLAG-Stat5 levels in each vertical gate, and the corresponding maximal p-Stat5 signal intensity (p-Stat5_max_), in wild-type cells (top panel, *R* ^2^ = 0.96) and EpoR-HM S3 cells (lower panel, *R* ^2^ = 0.998); analysis is of data shown in panel C. p-Stat5_max_ is the maximal p-Stat5 response to Epo, defined by the Hill equation in Figure S3 and obtained by fitting Hill curves to the data in (C).

To test whether the loss of the high-intensity p-Stat5 response in mature, S3 cells is indeed due to their decreased Stat5 expression (Figure 4D–F), we asked whether we could rescue high-intensity Stat5 signaling in these cells by exogenously expressing Stat5. In parallel, we also examined the effect of exogenous Stat5 expression in EpoR-HM erythroblasts, which signal exclusively via the low-intensity binary signaling mode (Figure 3C–E). We electroporated FLAG-tagged Stat5a constructs (“FLAG-Stat5”), or two control constructs, either FLAG-tagged Stat5aY694F (“FLAG-Stat5Y694F”) lacking the C-terminal tyrosine, or “empty vector” (“pcDNA3”), into freshly isolated wild-type or EpoR-HM fetal liver. Cells were incubated overnight in the presence of Epo (0.2 U/ml) to allow expression of the transduced constructs and were then deprived of Epo for 3 h prior to stimulation with a range of Epo concentrations (0.004 to 9 U/ml) for 15 min. Cells were immediately fixed and labeled with both anti-FLAG and anti-p-Stat5 antibodies. A single electroporation contained cells with a spectrum of FLAG expression levels, allowing us to determine how FLAG-Stat5 expression affected the p-Stat5 response (Figure 5).

We first examined how exogenous FLAG-Stat5 protein levels compared with endogenous Stat5 (Figure S9). Freshly isolated S3 cells express lower levels of the Stat5 protein than S1 cells (Figure 4D,E, Figure S9A, top panel). Following transfection with FLAG-Stat5, Stat5 protein in S3 cells increased to levels similar to those of the endogenous protein in S1 cells (Figure S9A, lower panel). We were therefore in a position to ask whether increasing Stat5 protein in S3 would be sufficient for these cells to generate the high-intensity p-Stat5 signal characteristic of S1. A minority of transfected S3 (18%, Figure S9A) expressed FLAG-Stat5 at higher levels than endogenous Stat5 in fresh S1. Of these, approximately 2% retained p-Stat5 following 3 h Epo deprivation (Figure 5A, double-headed arrow). We excluded all cells expressing the very high FLAG-Stat5 levels from further analysis, by gating specifically on cells with lower FLAG fluorescence. This was possible as FLAG fluorescence was an accurate measure of the level of the exogenous FLAG-Stat5 protein (Figure S9B).

For a given Epo concentration, the p-Stat5 response of transfected S3 cells increased with increasing FLAG-Stat5 levels (Figure 5A, top middle panel). There was no increase in the p-Stat5 signal in cells transfected with FLAG-Stat5Y694F, verifying that the p-Stat5 signal detected with increasing FLAG-Stat5 is indeed specific (Figure 5A, central panel). To analyze the p-Stat5 response quantitatively for each FLAG-Stat5 expression level, we sub-divided the “p-Stat5 versus FLAG-Stat5” dot histograms into narrow vertical gates, each containing cells with similar levels of FLAG-Stat5 (Figure 5B, left panels). Three of these vertical gates, numbered 10 to 12, are color coded in red, green and blue respectively. Cells in these gates are shown either unstimulated (Figure 5B, top left panel) or stimulated with Epo concentrations of 0.33 U/ml (middle left panel) or 9 U/ml (Figure 5B, lower left panel). Panels to the right show an overlay of the cells' responses in each of the red, green, or blue vertical gates (Figure 5B).

The entire dataset of the p-Stat5 response to nine Epo concentrations in each of four vertical gates (9 to 12) for either wild-type or EpoR-HM S3 cells were fitted with Hill curves (Figure 5C). These show that exogenous FLAG-Stat5 has two principal effects. First, the maximal response (p-Stat5_max_) in any given vertical gate is positively and linearly correlated with the level of FLAG-Stat5 protein in that gate (Figure 5D). Second, as FLAG-Stat5 levels increase, there is a decrease in the steepness of the p-Stat5 response curve, reflected by a decreasing Hill coefficient (Figure 5C). As examples, transfected EpoR-HM S3 cells containing high FLAG-Stat5 levels had a dose/response curve with a lower Hill coefficient and a higher p-Stat5_max_ (n_H_ = 1.0, p-Stat5_max_ = 1,100, gate 12, FLAG MFI = 1,700) than cells in the same sample containing lower levels of FLAG-Stat5 (n_H_ = 4.0, p-Stat5_max_ = 120, gate 9, FLAG MFI = 340). Similarly, wild-type S3 cells containing very low levels of FLAG-Stat5 had a dose/response curve with a higher Hill coefficient and lower p-Stat5_max_ (n_H_ = 3.5, p-Stat5_max_ = 25, gate 6, FLAG MFI = 75) than cells in the same sample with higher FLAG-Stat5 (n_H_ = 1.0, p-Stat5_max_ = 100, gate 8, FLAG MFI = 200).

Therefore, by varying the level of the Stat5 protein in mature S3 erythroblasts from either wild-type or EpoR-HM fetal livers, we were able to generate the entire spectrum of Stat5 signaling responses encountered in the erythroblast maturation series (Figure 1E). Taken together, the data in Figures 4 and 5 show that decreasing Stat5 protein levels with erythroblast maturation is the cause of the gradual shift from high-intensity, graded signaling in early erythroblasts to low-intensity, binary signaling in mature erythroblasts.

The loss of high-intensity Stat5 signaling in EpoR-HM shows that, in addition to high levels of the Stat5 protein, this mode of signaling also requires Stat5 phosphotyrosine docking sites on the EpoR. Exogenous expression of Stat5 successfully compensated for the EpoR-HM mutation, rescuing high-intensity graded signaling in these cells (Figure 5).

The linear dependence of p-Stat5_max_ on Stat5 protein levels, whether endogenous (Figure 4F) or exogenous (Figure 5D), indicates that Stat5 is limiting for Stat5 phosphorylation in erythroid cells. By contrast, EpoR expression in erythroblasts is not limiting to the maximal p-Stat5 response (Figure 4C). The Michaelis-Menten model of enzyme kinetics assumes that the substrate is present in excess. It therefore is unlikely to apply to the behavior of Stat5 activation in erythroblasts [31],[32]. The kinetics that apply instead is further analyzed in Text S1.

### Binary Low-Intensity Stat5 Signaling Rescues Mice from Fatal Perinatal Anemia

**Figure 6:**
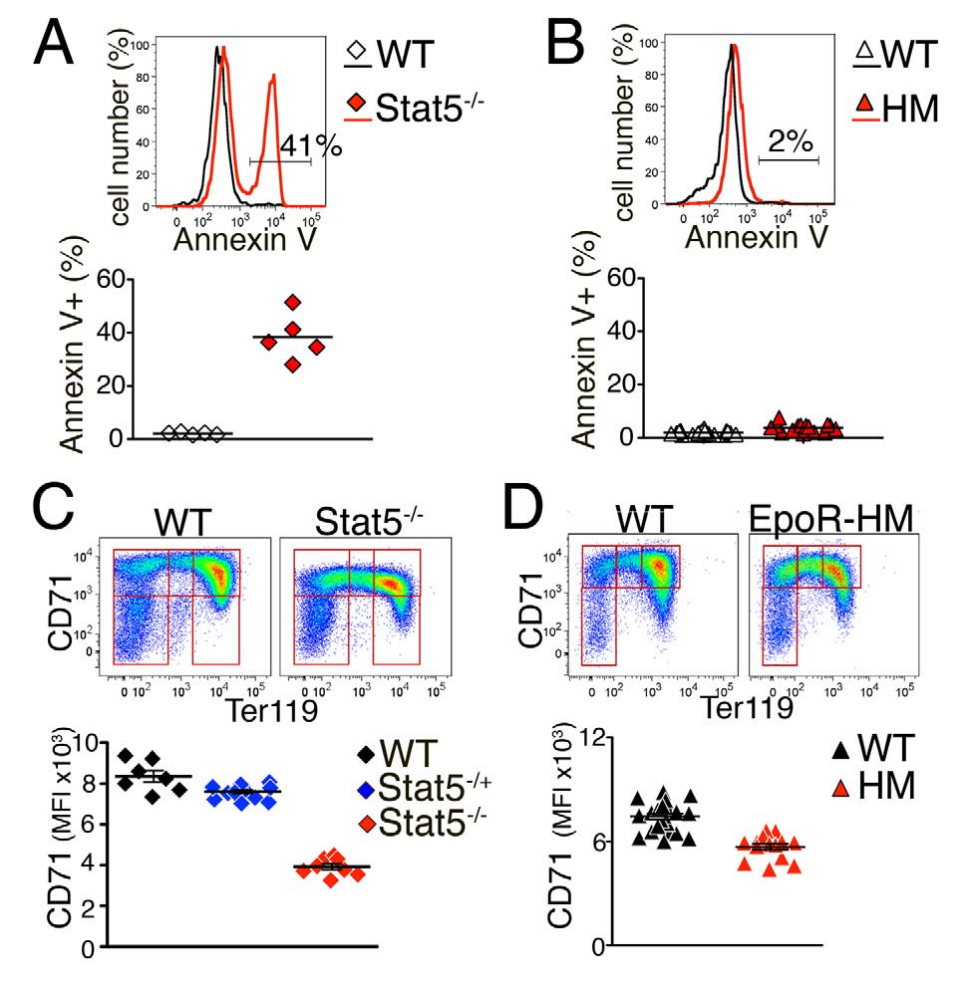
Stat5 functions in EpoR-HM and Stat5^−/−^ fetal liver. (A, B) Increased erythroblast apoptosis in Stat5^−/−^ but not in EpoR-HM fetal liver. Freshly harvested fetal livers from E13.5 Stat5^−/−^ or EpoR-HM embryos and from littermate or strain-matched controls were incubated in the absence of Epo for 90 min. Cells were then labeled with 7-AAD to exclude dead cells, and with Annexin V, CD71, and Ter119 to assess apoptosis. Representative histograms are shown for S1 cells in Stat5^−/−^ fetal liver and in matched control (A), and for S1 cells of EpoR-HM fetal liver and matched control (B). Summary of Annexin-V^+^ cells in three independent experiments is shown in corresponding lower panels; each data point corresponds to an individual embryo, the black line is the mean for all embryos of a given genotype. Number of embryos analyzed: EpoR-HM = 15, strain and age matched controls = 20; Stat5^−/−^ = 5, littermate controls = 5. No statistically significant difference was detected between EpoR-HM and control embryos; the difference between Stat5^−/−^ and control embryos is significant (*p* = 0.0007, two-tailed *t* test, unequal variance). (C, D) Cell-surface CD71 in Stat5^−/−^ and EpoR-HM fetal liver. E13.5 or E14.5 fetal liver from Stat5^−/−^, EpoR-HM, or matched control embryos were labeled with CD71 and Ter119. Non-viable cells were excluded with 7-AAD. Top panels show representative flow-cytometric CD71/Ter119 profiles of viable, non-fixed fetal liver cells. Lower panels show data for all Stat5^−/−^ embryos (*n* = 7, each data point is an individual embryo), Stat5^+/−^ (*n* = 11), and wild-type littermates (*n* = 8), and for EpoR-HM (*n* = 19) and strain-matched controls (*n* = 14). Difference between HM and matched control is significant (*p*<0.002, unpaired *t* test); Differences between Stat5^+/−^, Stat5^−/−^, and wild-type mice are all significant (*p*<0.0001, one-way ANOVA).

We used Stat5^−/−^ and EpoR-HM mice to elucidate the specific biological functions of the binary and graded Stat5 signaling modalities. Mice lacking Stat5 die perinatally of severe anemia [6],[12],[33],[34], suggesting that the functions of Stat5 in erythropoiesis are essential to life. By contrast, EpoR-HM mice, which retain only the binary low-intensity p-Stat5 signal, are viable and have near-normal basal erythropoiesis. Therefore, the low-intensity binary p-Stat5 signal is sufficient to support the essential erythropoietic Stat5 functions required for life. We examined this further by measuring Epo-mediated anti-apoptotic signaling in Stat5^−/−^ and EpoR-HM fetal liver erythroblasts. Anti-apoptosis is a key function of Epo-activated Stat5 in both basal and stress erythropoiesis, and is mediated by its transcriptional activation of the anti-apoptotic protein bcl-x_L_ and other targets [5]–[7],[34],[35]. We incubated fetal liver cells freshly isolated from EpoR-HM, Stat5^−/−^ and strain- matched wild-type control embryos in the absence of Epo for 90 min. We then labeled the cells with Annexin V, to detect cells undergoing apoptosis (Figure 6A,B). As reported previously [6],[34], a large fraction (40%) of Stat5^−/−^ S1 cells, but only 1%–2% of wild-type controls, were Annexin V positive, confirming the essential role for Stat5 in erythroblast survival (Figure 6A). By contrast, there was little apoptosis in the EpoR-HM fetal liver (Figure 6B, representative example in upper panel, summary of embryo litters in lower panel). Therefore, the low-intensity, binary Stat5 signal generated in EpoR-HM erythroblasts is sufficient for mediating Stat5's anti-apoptotic functions.

### EpoR-HM Adult Mice Fail to Upregulate Erythroblast CD71, a Target of EpoR Stress Signaling

**Figure 7:**
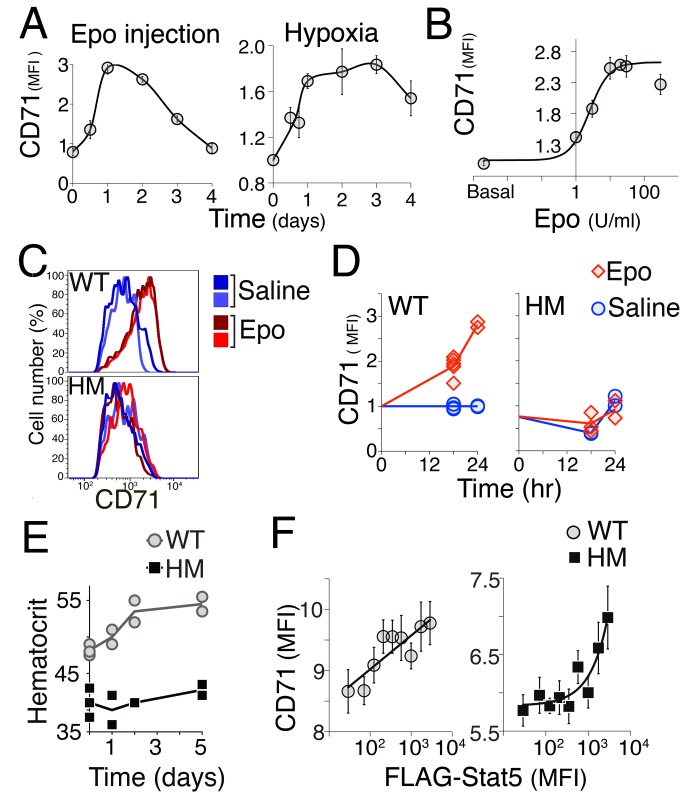
CD71 up-regulation is an EpoR-activated stress response that requires the high-intensity, graded p-Stat5 signal. (A) Erythropoietic stress causes up-regulation of cell-surface CD71 on spleen EryA erythroblasts (CD71^high^Ter119^high^FSC^high^). At t = 0, Balb/C mice were injected with Epo subcutaneously (300 U/ml, left panel) or placed in an hypoxia chamber (11% oxygen, right panel). Spleen was harvested at the indicated time points. Cell-surface CD71 was measured using flow-cytometry in EryA. Data points are MFI ± sem for three to six mice per time point, expressed as relative fluorescence units. (B) A dose/response curve in vivo of CD71 up-regulation in response to stress levels of Epo. Balb/C mice were injected with a single subcutaneous Epo dose as indicated. Spleen was harvested at t = 24 h post-injection, and EryA CD71 was measured by flow cytometry. Data points are MFI ± sem of three to eight mice per Epo dose, pooled from two independent experiments, and expressed relative to CD71 levels at t = 0. Bone-marrow EryA CD71 increased similarly. (C–E) EpoR-HM mice fail to up-regulate CD71 and to increase erythropoietic rate in response to stress levels of Epo. Adult EpoR-HM mice or strain-matched wild-type mice were injected with a single Epo dose (100 U/25 g mouse) or with saline control. Cell surface CD71 (C, D) and hematocrit (E) were measured at the indicated time points. (C) Representative CD71 flow-cytometry histograms of spleen EryA, in either wild-type (WT) or EpoR-HM (HM) mice, injected with either saline or Epo, at 24 h post-injection. Two mice are shown for each condition. (D) Summary of data measured as in (C), for HM and WT mice injected with either Epo or saline, at 18 and 24 h post-injection. Each datapoint represents CD71 levels (MFI) in spleen EryA from a single mouse. Two to five mice are shown per time point/genotype combination. (E) Hematocrit response to Epo injection. The hematocrit increases in the wild-type, but not in the EpoR-HM mouse. (F) Rescue of stress-induced CD71 up-regulation in EpoR-HM fetal liver cells after rescue of high-intensity graded p-Stat5 signaling by transduction with high levels of FLAG-Stat5. EpoR-HM or wild-type fetal liver erythroblasts were transduced with either FLAG-Stat5 or FLAG-Stat5Y694, as described in Figure 5A. Cells were incubated overnight in the presence of stress-levels of Epo (0.2 U/ml; this is 6- to 10-fold the Epo basal levels), and were then labeled and analyzed for CD71, Ter119, and FLAG expression by flow-cytometry. Datapoints are CD71 (MFI ± sem) for each FLAG vertical gate, as illustrated in Figure 5B, following correction for background fluorescence by subtracting the corresponding fluorescence for each FLAG gate of samples transduced with FLAG-Stat5Y694F.

Although adult EpoR-HM mice are viable, they are nevertheless mildly anemic, and are deficient in their response to erythropoietic stress [3],[36]. Given our finding that these mice retain the binary but lack the graded high-intensity Stat5 signaling mode, we asked whether the latter is specifically required during stress. The transferrin receptor, CD71, was recently identified as a Stat5 transcriptional target, and Stat5^−/−^ fetal liver erythroblasts were found to express 50% lower levels of cell-surface CD71 (Figure 6C; [12],[34]). Here we found that EpoR-HM fetal liver erythroblasts had a milder, though statistically significant, 15% loss of CD71 expression (Figure 6D; *p*<0.002, unpaired *t* test), potentially the result of their Stat5 signaling deficit. Although CD71 is highly expressed on fetal and adult erythroid progenitors during basal erythropoiesis, we found that there is a substantial, further increase in its cell-surface expression during the stress response (Figure 7A). Thus, a single subcutaneous Epo injection, which generates stress levels of Epo in blood for ∼24 h, caused a 3-fold increase in CD71 on the surface of splenic EryA erythroblasts (CD71^high^Ter119^high^FSC^high^ [21]) (Figure 7A, left panel). Further, CD71 increased nearly 2-fold in the same cells in mice placed in a reduced oxygen environment (11% oxygen, Figure 7A, right panel); plasma Epo in these mice rises ∼3-fold in the initial 3 days following the onset of hypoxia. An in vivo Epo dose/CD71 response analysis showed a graded increase in cell surface CD71 in response to increasing Epo, with half the maximal increase seen in mice injected with 3 U of Epo/25 g body weight (Figure 7B), and a Hill coefficient of 1.5. These findings establish CD71 as a target of erythropoietic stress whose level is modulated with the degree of stress.

Given the mild but significant deficit of CD71 expression in EpoR-HM fetal liver erythroblasts (Figure 6D), we examined expression of erythroblast CD71 during the response of EpoR-HM adult mice to stress (Figure 7C,D). We found that, unlike wild-type mice, EpoR-HM mice completely failed to upregulate CD71 when injected with high Epo (100 U/25 g mouse; Figure 7C,D). This failure may account in part for the failure of EpoR-HM mice to accelerate erythropoiesis and increase their hematocrit (Figure 7E) [3].

### Exogenous Stat5 Rescues Stress-Induced CD71 Up-Regulation in EpoR-HM Erythroblasts

Since high exogenous Stat5 restored the high-intensity graded Stat5 signaling missing in EpoR-HM erythroblasts (Figure 5C), we asked whether it may also restore high CD71 expression. We measured CD71 expression in EpoR-HM and wild-type fetal liver cells that were electroporated with FLAG-Stat5 in the experiment illustrated in Figure 5A–D, following overnight culture in stress Epo levels (0.2 U/ml, Figure 7F). We found that cells with increasing FLAG-Stat5 protein showed a corresponding, gradual increase in cell-surface CD71, in both wild-type and EpoR-HM cells (Figure 7F). These findings strongly suggest that the graded, stress-dependent CD71 up-regulation is a function specifically mediated by the high-intensity graded Stat5 signal during the erythropoietic response to stress.

## Discussion

EpoR-activated Stat5 signaling in erythroid progenitors begins with the transition from S0 to S1 (Figure S1A), a transition that marks a developmental switch comprising transcriptional and epigenetic erythroid commitment events including the onset of dependence on EpoR signaling [18],[19]. We identified two modalities of p-Stat5 signaling in erythropoietic tissue, graded and binary, each with distinct biological functions, which together increase the information content of the Stat5 signal and allow differential regulation of basal and stress erythropoiesis. In early erythroblasts, a graded increase in Epo concentration generates a graded p-Stat5 signal that reaches high intensities in response to stress levels of Epo. The maximal p-Stat5 signal intensity declines, however, with erythroblast maturation, to a 4-fold lower level in more mature, S3 erythroblasts. The low-intensity p-Stat5 signal in S3 erythroblasts has a steep response to increasing Epo concentrations, characterized by Hill coefficients in the range of three to four, which is similar to or steeper than the cooperative binding of oxygen to hemoglobin [25]. This steepness converts a graded Epo input into a binary, “on” or “off” response.

The gradual loss of high-intensity p-Stat5 signaling with erythroid maturation is due in part to increasing expression of SOCS3 (Figure S6) and to declining Stat5 protein (Figure 4). The role played by Stat5 was first indicated by the strong correlation between Stat5 protein levels and the maximal p-Stat5 signal intensity, across all erythroblast differentiation subsets (Figure 4F). We used exogenous Stat5 to confirm a causal role for Stat5 protein levels in determining Stat5 signaling modality. Thus, we were able to endow mature erythroblasts expressing high levels of exogenous Stat5 with high-intensity graded signaling, and showed that maximal p-Stat5 signal intensity was proportional to exogenous Stat5 protein levels (Figure 5D).

### Distinct Biological Functions for the Binary and Graded Stat5 Signaling Modalities

The biological functions of the two Stat5 signaling modalities are exemplified by the EpoR-HM and Stat5^−/−^ mouse models. EpoR-HM erythroblasts signal exclusively via the binary low-intensity signal. Unlike Stat5^−/−^ mice, which die of fatal perinatal anemia due to erythroblast apoptosis, EpoR-HM mice are viable with near-normal basal erythropoiesis and normal erythroblast survival (Figure 6B). The binary low-intensity pStat5 signal conveys binary, life or death decisions that rescue sufficient numbers of erythroblasts from apoptosis to make developmental and basal erythropoiesis possible.

By contrast, the EpoR-HM mice lack an efficient stress response (Figure 7E, [3]). We found that up-regulation of CD71 on the surface of erythroid precursors is a stress-specific graded response that depends on high Epo levels in vivo (Figure 7A.B). It requires the graded, high-intensity p-Stat5 signal that is elicited by stress levels of Epo and that is missing in EpoR-HM mice. This is evident from the finding that EpoR-HM erythroblasts fail to up-regulate CD71 when subjected to high Epo and from rescue of the CD71 response in EpoR-HM erythroblasts transduced with exogenous Stat5, which restores the high-intensity p-Stat5 signal to these cells (Figure 7F, Figure 5C). Exogenous Stat5 similarly endowed mature wild-type erythroblasts with both high-intensity graded Stat5 signaling and with the ability to induce stress levels of CD71 (Figure 7F, Figure 5C). These findings strongly suggest that the ability of an erythroblast to generate the CD71 stress response is determined by its ability to generate the high-intensity p-Stat5 signal, and not by other aspects of erythroblast maturation.

### The Transferrin Receptor Is a Novel Target of Erythropoietic Stress

Although the transferrin receptor (CD71) is ubiquitous in dividing cells, it is expressed at uniquely high levels in erythroid progenitors, where it provides the high iron requirement for hemoglobin synthesis. Genetic mutations that decrease either CD71 or plasma iron compromise erythropoiesis, resulting in anemia and a loss of the stress response [37],[38]. Recently, Stat5 was shown to be required for optimal erythroblast CD71 expression in the fetus [12],[34].

Here we found that, during stress, CD71 in early erythroblasts increases beyond its already high level in basal erythropoiesis (Figure 7A–B). This increase is a Stat5-dependent function that specifically requires the high-intensity Stat5 signaling mode (Figure 7C–F). Though not reported previously, the increase in cell-surface transferrin receptor during stress is consistent with the higher requirement for iron during stress erythropoiesis [37],[38]. It is also consistent with the increase in plasma soluble transferrin receptor, a known clinical indicator of increased erythropoietic rate [39]. The failure of EpoR-HM mice to up-regulate CD71 may therefore account for their deficient stress response. It is likely, however, that additional functions regulated by the high-intensity p-Stat5 signal also contribute, including a stress-dependent increase in the level of the anti-apoptotic bcl-x_L_ protein [5].

### Coexisting Graded and Binary Modalities Permit High Fidelity Signaling over a Wide Epo Range

Binary and graded signaling modes have fundamentally different functional consequences. The steepness of the binary dose/response curve has the advantage of filtering out noise and generating a clear signal that is easily distinguishable from background. This mode of signaling is therefore ideal at the low end of the Epo concentration range, where Epo stimuli, though low, are nevertheless essential for basal erythropoiesis and must be clearly distinguished from noise. A key disadvantage of binary signaling, however, is its inability to encode incremental changes in stimulus. This would exclude it as a useful signaling modality in erythropoietic stress, where Epo concentration determines the precise level of erythropoietic acceleration that is required to compensate for hypoxia.

**Figure 8:**
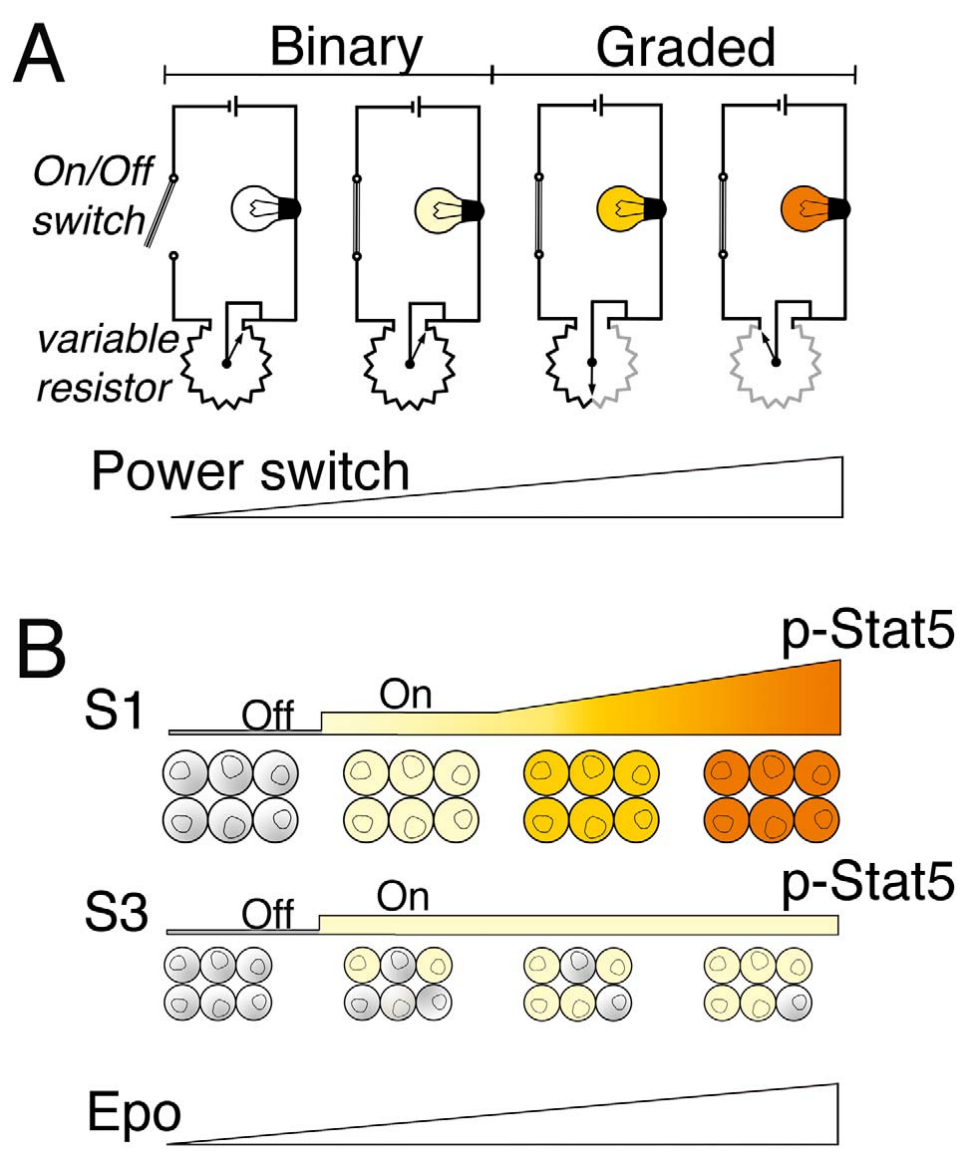
Dimmer-switch model of Stat5 signaling. (A) The operation of a dimmer switch combines binary and graded components. The closing of an “on/off” switch closes an electric circuit, allowing current to flow and switches on a dim light. A gradual further turning of the power-switch dial permits a gradual decrease of the circuit's resistance, with a consequent graded increase in the electric current and light intensity. (B) The p-Stat5 signal is turned “on” through a binary action, to produce a low-intensity but decisive signal in S3 erythroblasts or in EpoR-HM erythroblasts. In S1 early erythroblasts, this signal can increase further with a gradual further increase in Epo concentration. No further increase takes place in mature S3 erythroblasts, though the number of signaling erythroblasts increases with increasing Epo.

Stat5 bridges this conundrum by combining the binary and graded signaling modalities in a manner analogous to a dimmer switch (Figure 8A), allowing signaling fidelity over a wide Epo concentration range. Low stimuli activate the binary component of the dimmer switch from “off” (open on/off switch, Figure 8A) to “on” (closed on/off switch), which closes the electric circuit and switches the light on. A further turning of the power dial incrementally reduces the circuit's resistance, resulting in an incremental, graded increase in light intensity. Similarly, low Epo stimuli result in a binary activation of p-Stat5. In S1 cells, this binary activation is superseded at higher Epo stimuli with a further, graded increase in the p-Stat5 signal intensity (Figure 8B). Of note, although S3 cells are individually limited to a low-intensity binary response, increasing Epo results in an increasing number of signaling S3 cells, due to their varying activation thresholds (Figure 8B).

At the level of gene transcription, p-Stat5 signal intensity, rather than the steepness of the dose/response curve, is likely to determine which subset of gene targets will be activated. As example, it is likely that activation of lower affinity Stat5 binding sites will require higher p-Stat5 concentration, manifesting as higher signal intensities. Further, the p-Stat5 signal intensity may affect the likelihood of formation of Stat5 tetramers, which appear to bind to a functionally distinct subset of Stat5 targets [40],[41].

While a steep dose/response curve is unlikely to determine which Stat5 targets are activated, its role is to ensure that the low-intensity p-Stat5 signal is generated only in response to a biologically appropriate stimulus. A key challenge of low-intensity signals is their inherently low signal-to-noise ratio. The steep dose/response curve of binary signaling provides a threshold for activation that filters out random noise and ensures that the low-intensity signal is decisive.

Networks containing similar signaling components and similar topologies may vary in their output, generating either binary (digital) or graded (analog) responses, depending on the value of key parameters [42]–[44]. Thus, apparently homologous MAPK modules generate a switch-like response in Xenopus oocytes, but a graded response in the yeast mating pheromone pathway [43]. Further, isogenic yeast cells use a similar complement of transcription factors to generate either binary or graded transcriptional responses from the Gal1 promoter [42]. The mechanism(s) that determine whether a response is binary or graded in these examples is not fully understood [43]. By contrast, Ozbudak et al. showed both theoretically and experimentally that it is possible to interconvert binary and graded responses of the E. coli lac operon simply by titrating the Lac repressor LacI [44]. Recent reports suggest that binary and graded signaling modalities may coexist in cells [16],[45]. Thus, Ras signaling in T lymphocytes is of a low-intensity, analog form, but can assume a high-intensity, digital form when an SOS positive feedback loop is activated.

Here we suggest that low concentration of the Stat5 protein results in a binary response, while a high concentration generates a graded response. This model is consistent with the following data: (i) steeper dose/response curves in the Stat5^+/−^ S3, which contain less Stat5, compared with wild-type S3 (Figure 4B); (ii) steeper dose/response curves in the more mature subsets of S3 (Figure 3G), consistent with decreasing levels of Stat5 protein with maturation (Figure 4D–E); and (iii) gradual conversion from binary to graded responses in cells expressing increasing levels of transduced FLAG-Stat5 (Figure 5C).

The binary response of EpoR-HM may be explained within the same framework. Presumably, docking of Stat5 on EpoR phosphotyrosines increases Stat5 concentration in the immediate vicinity of the EpoR/Jak2 complex. The absence of EpoR phosphotyrosines in EpoR-HM might be expected to result in lower Stat5 concentration within the locality of EpoR/Jak2 and be functionally equivalent to low cellular Stat5. Support for this comes from the fact that we can rescue graded signaling in EpoR-HM by transducing these cells with high FLAG-Stat5 (Figure 5C, lower panels).

It is unclear at this point how Stat5 concentration determines the steepness of the dose/response curve, a question that will form the focus of future work.

### EpoR Does not Limit Stat5 Signaling Intensity in Erythroblasts

Reduced levels of EpoR in EpoR^+/−^ erythroblasts do not prevent the generation of a maximal p-Stat5 signal (Figure 4C, Figure S8). Further, the low-intensity p-Stat5 signal can be converted into a high-intensity signal by exogenous high expression of FLAG-Stat5, consistent with EpoR expression in these cells not being limiting to the p-Stat5 signal (Figure 5C–D).

Estimates of the EpoR cell-surface occupancy required to generate the p-Stat5 response are consistent with the conclusion that cell surface EpoR is not limiting for this response. Using a value of 130,000 U of Epo per milligram [46], a dissociation constant (K_D_) for Epo of 240 pmol/L [1] and Epo's molecular weight (34,000 Daltons), 50% occupancy will be seen at Epo concentrations of 1 U/ml. This is a much higher concentration than the apparent K_m_ for generating the half-maximal p-Stat5 response, which we found to be between 0.06 and 0.15 U/ml (Figures S3B, S7A, S7C). Assuming a hyperbolic binding curve for Epo, basal Epo levels (0.010–0.020 U/ml) would occupy only 1%–2% of the cell surface EpoR, and an Epo concentration of 0.1 U/ml, generating half the maximal p-Stat5 response, would increase EpoR occupancy to 10%. At 35% occupancy, the p-Stat5 response is expected to be near-maximal in all erythroblast subsets. The very highest Epo levels, found for example in aplastic anemia, of 10 U/ml, result in 90% EpoR occupancy. This analysis suggests that cell-surface EpoR has vast reserves with respect to the generation of the p-Stat5 signal.

### The Role of Stat5 Dosage in Developmental and Disease-Related Stat5 Signaling

We found that the maximal p-Stat5 signal intensity generated by a maximal Epo stimulus is largely determined by Stat5 protein levels (Figures 4F, 5C–D), though it is also affected by high SOCS3 expression in mature erythroblasts (Figure S6).

Michaelis-Menten enzyme kinetics assumes that the substrate is present in excess, and is therefore not applicable to Stat5 signaling in erythroblasts, where the substrate is limiting ([31],[32]; see Text S1). This non-Michaelian behavior may explain recent reports linking higher Stat5 gene dosage or expression to leukemogenesis [47],[48]. Thus, based on our findings, we suggest that the higher Stat5 protein found in leukemia cells may be causing a higher p-Stat5 signal, possibly activating gene targets that contribute to leukemogenesis. These considerations underscore the importance of identifying regulators of Stat5 expression both during normal erythroid differentiation and in leukemia.

Our findings raise the possibility that there may be signaling pathways other than EpoR-Jak2-Stat5 in which the second messenger molecule, and not its upstream receptor, is limiting to the signal response. This non-Michaelian behavior has implications when such pathways are activated pathologically. To date, inhibition of abnormal signaling in tumor cells has largely focused on membrane or nuclear receptors and on other early or first steps of signaling cascades. Examples include the inhibition of the epidermal growth-factor (EGF) receptors, over-expressed in many solid tumors, and inhibition of Jak2 or Bcr-Abl in myeloproliferative disease and leukemia [49]–[52]. Our work suggests an alternative therapeutic paradigm, in which targeting second messengers that are limiting to signal transduction may be an effective therapeutic strategy. In the case of Stat5, targeting its high-intensity signaling may inhibit its function in myeloproliferative disease without affecting the binary low-intensity p-Stat5 response in normal cells.

## Materials and Methods

### Fetal Liver Cell Preparation

Fetal livers were isolated at E12.5–E14.5, dissociated mechanically, and deprived of Epo for 90 min in the presence of 20% serum prior to Epo stimulation. Electroporations were performed using Amaxa Biosystem Nucleofector on fresh fetal liver. Cells were incubated for 18 h in Epo (0.2 U/mL), Stem Cell Factor (100 ng/mL), and Interleukin-3 (10 ng/mL) and washed 3 times and incubated in 20% serum for 3 h prior to Epo stimulation.

### Flow Cytometry

Epo-stimulated cells were harvested in phosphowash (PBS, 1 mM sodium orthovanadate, 1 mM β-glycerol phosphate, 1 µg/mL microcystin), fixed in 1.6% paraformaldehyde, permeabilized in 80% acetone, and stored at −80°C. Thawed cells were stained in PBS/3% milk with AF647-conjugated anti-phospho Stat5 (612599, BD Biosciences), for Ter119 and CD71 as described [21], and where indicated, for Stat5 (ab 7969, Abcam followed by anti-rabbit-APC, Jackson ImmunoResearch Laboratories), FLAG (F4049, Sigma Aldrich), and Myc (2272, Cell Signaling Technology). In all electroporation experiments, cells were stained with LIVE/DEAD Fixable Blue Dead Cell Stain Kit for UV excitation (L-23105, Invitrogen), prior to fixation and permeabilization in order to exclude dead cells from analysis. λ-phosphatase treatment was for 15 min at 37°C on fixed and permeabilized cells (1,000 units, New England Biolabs).

Apoptosis assays were done on fresh fetal livers that were deprived of Epo for 90 min and then stained for CD71, Ter119, and Annexin V according to the manufacturer's instructions (BD Biosciences). Spleen and bone marrow cells isolated from adult mice were immediately stained with CD71 and Ter119 as described [21]. Cells were analyzed on an LSRII cytometer (BD Biosciences). Data were analyzed with FlowJo software (Tree Star, Stanford University, Stanford, CA).

For mouse strains, DNA constructs, quantitative RT-PCR, and si-RNA, see Text S2.

## Supporting Information

Figure S1 The p-Stat5 response in fetal liver. (A) The p-Stat5 response of fetal liver subsets S0 to S3 to Epo (2 U/ml, 15 min). Freshly isolated fetal liver cells were deprived of Epo for 90 min and were then stimulated. Cells were labeled for CD71, Ter119, and p-Stat5. (B) Time-course of the p-Stat5 response to Epo (2 U/ml for up to 6 h). Each of the three measures used to assess the p-Stat5 response (see Figure 1C, main text) is plotted versus time. Representative of five similar experiments. (C) Representative responses of S0 cells to stimulation with a range of Epo concentrations for 15 min. Flow-cytometry histogram overlay is shown. Even at the highest Epo concentrations, only 20% to 30% of S0 cells are responsive to Epo. For responding cells, the p-Stat5 MFI increases with Epo concentration, in the manner seen for S1 cells.
(TIF)

Figure S2 The p-Stat5 response in adult spleen and bone marrow in vivo. (A–F) Time course of the p-Stat5 response to Epo stimulation in vivo, in adult mouse bone marrow and spleen. Mice were injected with Epo (100 U/25 g mouse subcutaneously). Bone marrow and spleen were harvested at the indicated time points for up to 16 h following injection, and cells were immediately fixed, permeabilized, and labeled for CD71, Ter119, and p-Stat5. Erythroid subsets in adult mouse bone marrow or spleen may be defined by flow cytometry using cell surface Ter119 and CD71 [21]. Subsets ProE→EryA→EryB→EryC contain erythroid precursors of increasing maturity. The maturation stage of ProE resembles that of S2 in fetal liver and the maturation stage of EryA resembles that of S3. (A–C) Bone marrow subsets. (D–F) Spleen subsets. (A, D) The “total p-Stat5” response. (B, E) p-Stat5^+^ cells. (C, F) p-Stat5 in p-Stat5^+^ cells (solid lines). For comparison, the dashed lines show the “total p-Stat5” response data from (A, D), respectively. Data were pooled from four independent experiments. Each time point is the mean ± sem of data from two to four mice. MFI data are normalized as follows: background MFI in the absence of Epo is subtracted, and the remainder MFI is expressed as a ratio to MFI of p-Stat5^+^ cells in bone marrow EryA at time = 1 h for each experiment.
(TIF)

Figure S3Binary p-Sta5 signaling in EpoR-HM erythroblasts. (A) Representative plots of “total p-Stat5 versus Epo concentration” for subsets S1 to S3 in wild-type (left panel) and EpoR-HM (right panel) fetal liver. The same experiment as in Figure 3C, main text. (B) Three independent experiments assessing p-Stat5 signaling in EpoR-HM mice. Values for p-Stat5_max_ and apparent K_m_ were obtained by fitting Hill curves to plots of “total p-Stat5 MFI versus Epo concentration” of the type illustrated in (A). The Hill equation was used as follows: 
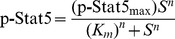
, where *S* = Epo concentration in U/ml, and p-Stat5 is the total p-Stat5 fluorescence; best fit was obtained by varying *n* ( = Hill coefficient, “n_H_” in the text), *K_m_* ( = the apparent K_m_), and p-Stat5_max_ ( = the maximal p-Stat5 response to high Epo), using the solver function of Microsoft Excel. *R* ^2^ is Pearson's product moment correlation coefficient, correlating experimental data with values predicted by the Hill equation for the corresponding Epo concentrations. The Hill coefficients and *R* ^2^ values for this analysis are shown in Figure 3E, main text. The K_m_ and p-Stat5_max_ were all significantly different in wild-type and EpoR-HM mice (paired *t* test, *p* = 0.003 and 0.023, respectively).(TIF)

Figure S4 Comparison of dose/response curves in E12.5 and E14.5 embryos. E12.5 and E14.5 fetal livers were stimulated with Epo and analyzed by flow cytometry in the same experiment. The data for the E14.5 fetal liver are also shown in Figure 3F–G. (A) CD71/Ter119 profiles of E12.5 and E14.5 fetal livers (left panels). The S3 subsets were divided into serial FSC gates, each corresponding to 300 channels (middle panels). Right panels show an overlay of Ter119 expression in FSC gates #3 and #6 for each of the embryos. (B) Epo dose/p-Stat5 response curves for the S3 FSC gates in the E12.5 and E14.5 embryos. Gate number and corresponding Hill coefficient are shown for each curve. (C) Overlay of the indicated dose/response curves.
(TIF)

Figure S5 Stat5 signaling in Shp1^−/−^ (C57BL/6J-Ptpn6^me^/J) fetal liver. (A) Quantitative real-time PCR for Shp1 in sorted S0–S3 subsets from wild-type embryos (E12.5–E13.5). The left panel shows Shp1 mRNA relative to β actin (mean ± SE), calculated from three independent experiments. The right panel shows the ΔCt ± SE for each individual experiment. (B) Epo dose/p-Stat5 response analysis in Shp1^−/−^ fetal liver fitted with Hill curves. Data (mean) from two independent experiments, each with one embryo of each genotype. Normalization as in Figure 1E. (C) Time course of p-Stat5 response to Epo stimulation with 0.2 U/ml, in Shp1^−/−^ embryos (*n* = 5) and in matched controls (*n* = 3). Data points are measurements in individual embryos.
(TIF)

Figure S6 SOCS3 regulation of Stat5 signaling. (A) Quantitative real-time PCR for SOCS3 in sorted S0–S3 subsets from wild-type embryos (E13–E14.5). The left panel shows SOCS3 mRNA relative to β actin (mean ± SE), calculated from three independent experiments. The right panel shows the ΔCt ± SE for each individual experiment. (B, C) Fetal liver cells were electroporated with SOCS3 siRNA or with “scrambled” control siRNA. Four hours later cells were stimulated with Epo. Both SOCS3 mRNA (B) and the p-Stat5 response (C) were measured at the indicated times.
(TIF)

Figure S7 p-Stat5 signaling in Stat5^+/−^ and EpoR^+/−^ erythroblasts. (A) Epo Dose/p-Stat5 response analysis of Stat5^+/−^ fetal liver. Values for the Hill coefficients, p-Stat5_max_, and apparent K_m_ were obtained by fitting Hill curves (see legend to Figure S3B) to the “total p-Stat5 MFI versus Epo concentration” data (main text, Figure 4B). Note a substantially lower p-Stat5_max_ in Stat5^+/−^ embryos. A total of seven Stat5^+/−^ and six control embryos were individually analyzed. *R* ^2^ is Pearson's product moment correlation coefficient. (B) EpoR mRNA in wild-type (WT, circles) and EpoR^+/−^ (squares) fetal liver, measured using quantitative RT-PCR at E13.5. Data points represent measurements in individual embryos, and are expressed relative to β actin mRNA. Mean values for each genotype are denoted with a black line. (C) Epo Dose/p-Stat5 response analysis of EpoR^+/−^ fetal liver. Analysis as for Stat5^+/−^ embryos in panel A, of data presented in Figure 4C. Note doubling of the apparent K_m_ in EpoR^+/−^ embryos (resulting in a shift of the curve to the right). A total of four EpoR^+/−^ and three control embryos were analyzed independently. (D) Measurements of Stat5 protein levels in fetal liver subsets using flow-cytometry. Fixed and permeabilized fetal liver cells were labeled with antibodies against Ter119 and CD71, and in addition, with a rabbit polyclonal antibody, which recognizes Stat5 regardless of its state of activation (ab 7969, Abcam) and a secondary anti-rabbit IgG antibody conjugated to APC. Flow cytometry histograms reflecting total Stat5 protein expression (as APC fluorescence) are shown for two Stat5^−/−^ embryos (these provide the non-specific background fluorescence), and for one wild-type and one Stat5^+/−^ embryo. Stat5 expression in Figure 4D–F was determined as Stat5 MFI, with background fluorescence subtracted, and expressed as a ratio to the average Stat5 MFI of S1 cells.
(TIF)

Figure S8 EpoR expression is not limiting to the maximal p-Stat5 signal. Ter119-negative cells were sorted from E13.5 fetal livers using magnetic beads. The cells from each fetal liver were divided into two aliquots, one of which was stimulated with Epo. The entire lysate from each aliquot was resolved using SDS-PAGE and analyzed by Western blotting. Two independent litters were processed. (A) Western blot analysis of embryos from a single litter. The membrane was stripped and re-probed sequentially with antibodies to p-EpoR, p-Stat5, and the transferrin receptor. The transferrin receptor signal indicates the presence of S1 erythroblasts. Chemiluminescence was quantitated using Bio-Rad Molecular Imager Chemi Doc XRS+ and Image Lab Software Version 3.0.1, Bio-Rad Laboratories. The pseudo-color is a software tool indicating increasing signal intensity, in the order pink ( = background), blue, green, and red ( = maximal). All signals were within the linear range as confirmed by sequential automated timed exposures. (B) The p-EpoR/p-Stat5 ratio for wild-type and EpoR^+/−^ embryos. Each data point is derived from a single fetal liver. Three wild-type and five EpoR^+/−^ fetal livers were pooled from two litters. Ratios were 1.7±0.02 for wild-type, 1.1±0.08 for EpoR^+/−^, *p* = 0.002, two-tailed *t* test with unequal variance.
(TIF)

Figure S9 Measurement of exogenous Stat5 protein in transfected fetal liver cells. (A, B) Fetal liver cells were electroporated with FLAG-tagged Stat5a (“FLAG-Stat5”) or with control “empty vector” (“pcDNA3”). Cells were incubated overnight in the presence of Epo (0.2 U/ml), to allow expression of the transduced constructs. Expression of exogenous FLAG-Stat5 protein and p-Stat5 signaling were measured at 18 h. (A) Stat5 protein levels are assessed as in Figure S7D. Top panel, Stat5 protein in freshly isolated S1 and S3 cells, prior to transfection with FLAG-Stat5. Middle panel, Stat5 protein in S3 cells transduced with either FLAG-Stat5 (red) or with empty vector (pcDNA3, green). Stat5 protein in fresh S3 cells (black) is shown for comparison. Lower panel, Stat5 protein in S3 cells transduced with FLAG-Stat5 (red), compared with Stat5 protein levels in fresh S1 cells (blue). (B) Measurement of FLAG fluorescence is an accurate assessment of exogenous Stat5 protein levels. S3 cells transfected with either FLAG-Stat5 (red) or empty vector (green), labeled with both anti-FLAG antibody (*x*-axis) and an anti-Stat5 antibody (*y*-axis). A linear correlation is observed between FLAG and Stat5 staining in FLAG-Stat5 transfected cells.
(TIF)

Text S1 Supplementary analysis: Cellular Stat5 is limiting during Stat5 phosphorylation in erythroid cells.
(PDF)

Text S2 Supplementary materials and methods.
(PDF)

## Abbreviations

Epo: erythropoietin
EpoR: Erythropoietin receptor
MFI: Median Fluorescence Intensity
n_H_: Hill coefficient
p-EpoR: phosphorylated EpoR
p-Stat5: phosphorylated Stat5
