# Supplementary figures and images for: Stat5 Signaling Specifies Basal versus Stress Erythropoietic Responses through Distinct Binary and Graded Dynamic Modalities
Source: PLoS Biol. 2012 Aug 28;10(8):e1001383. doi: 10.1371/journal.pbio.1001383 (PMC3433736; doi:10.1371/journal.pbio.1001383)

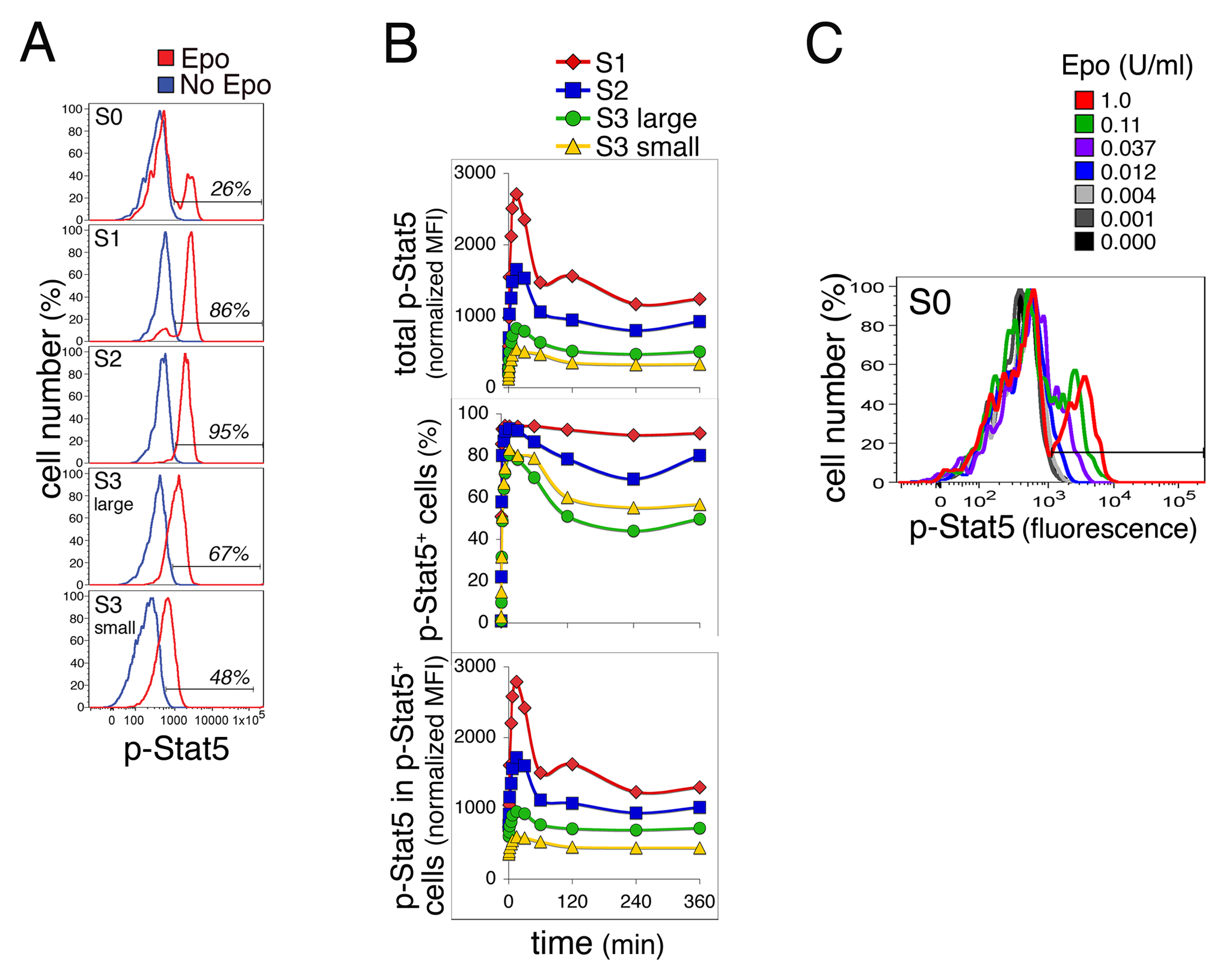

Supplement: Figure S1 — The p-Stat5 response in fetal liver. (A) The p-Stat5 response of fetal liver subsets S0 to S3 to Epo (2 U/ml, 15 min). Freshly isolated fetal liver cells were deprived of Epo for 90 min and were then stimulated. Cells were labeled for CD71, Ter119, and p-Stat5. (B) Time-course of the p-Stat5 response to Epo (2 U/ml for up to 6 h). Each of the three measures used to assess the p-Stat5 response (see Figure 1C, main text) is plotted versus time. Representative of five similar experiments. (C) Representative responses of S0 cells to stimulation with a range of Epo concentrations for 15 min. Flow-cytometry histogram overlay is shown. Even at the highest Epo concentrations, only 20% to 30% of S0 cells are responsive to Epo. For responding cells, the p-Stat5 MFI increases with Epo concentration, in the manner seen for S1 cells. (TIF) [file pbio.1001383.s001.tif]

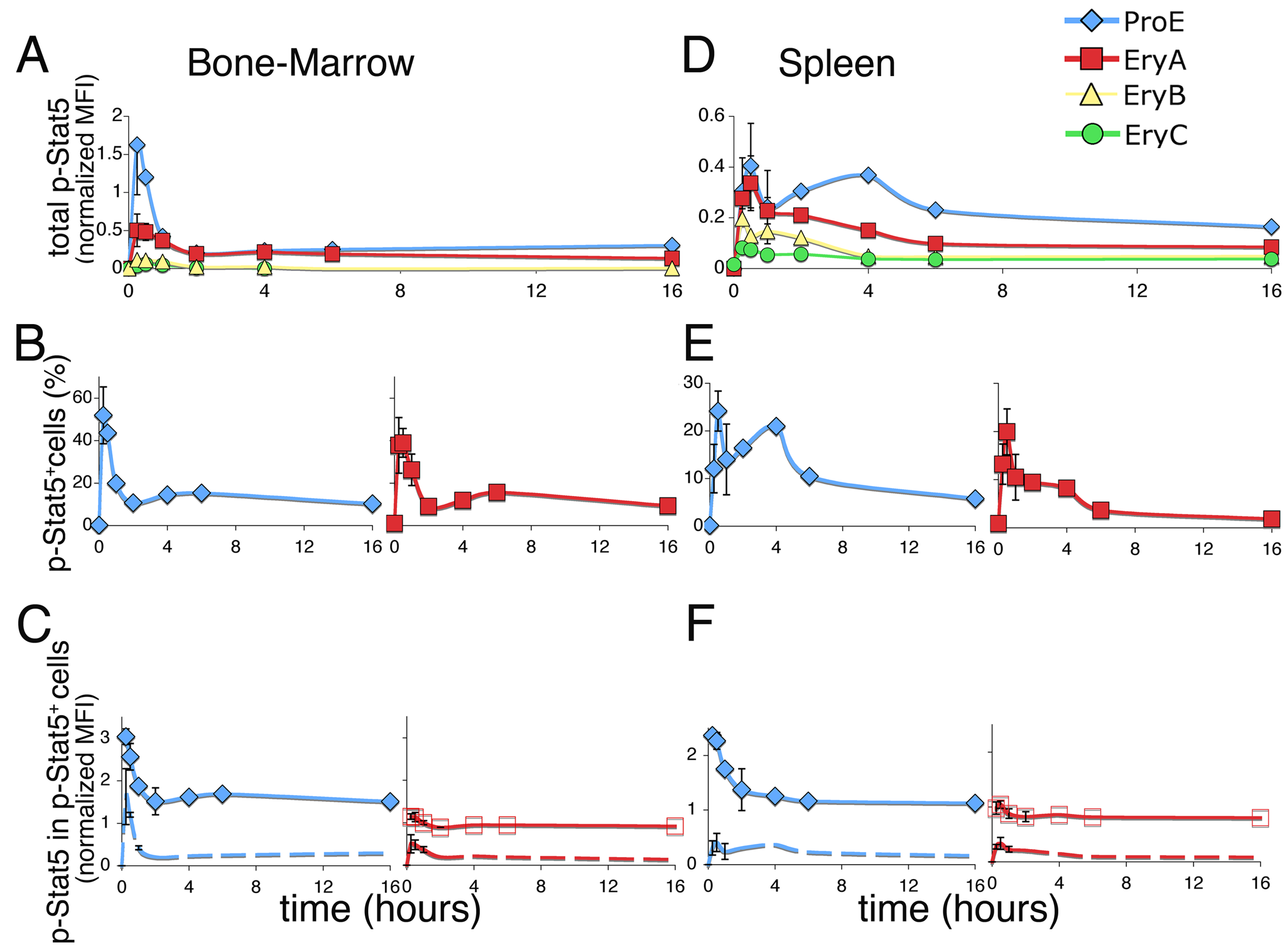

Supplement: Figure S2 — The p-Stat5 response in adult spleen and bone marrow in vivo. (A–F) Time course of the p-Stat5 response to Epo stimulation in vivo, in adult mouse bone marrow and spleen. Mice were injected with Epo (100 U/25 g mouse subcutaneously). Bone marrow and spleen were harvested at the indicated time points for up to 16 h following injection, and cells were immediately fixed, permeabilized, and labeled for CD71, Ter119, and p-Stat5. Erythroid subsets in adult mouse bone marrow or spleen may be defined by flow cytometry using cell surface Ter119 and CD71 [21]. Subsets ProE→EryA→EryB→EryC contain erythroid precursors of increasing maturity. The maturation stage of ProE resembles that of S2 in fetal liver and the maturation stage of EryA resembles that of S3. (A–C) Bone marrow subsets. (D–F) Spleen subsets. (A, D) The “total p-Stat5” response. (B, E) p-Stat5+ cells. (C, F) p-Stat5 in p-Stat5+ cells (solid lines). For comparison, the dashed lines show the “total p-Stat5” response data from (A, D), respectively. Data were pooled from four independent experiments. Each time point is the mean ± sem of data from two to four mice. MFI data are normalized as follows: background MFI in the absence of Epo is subtracted, and the remainder MFI is expressed as a ratio to MFI of p-Stat5+ cells in bone marrow EryA at time = 1 h for each experiment. (TIF) [file pbio.1001383.s002.tif]

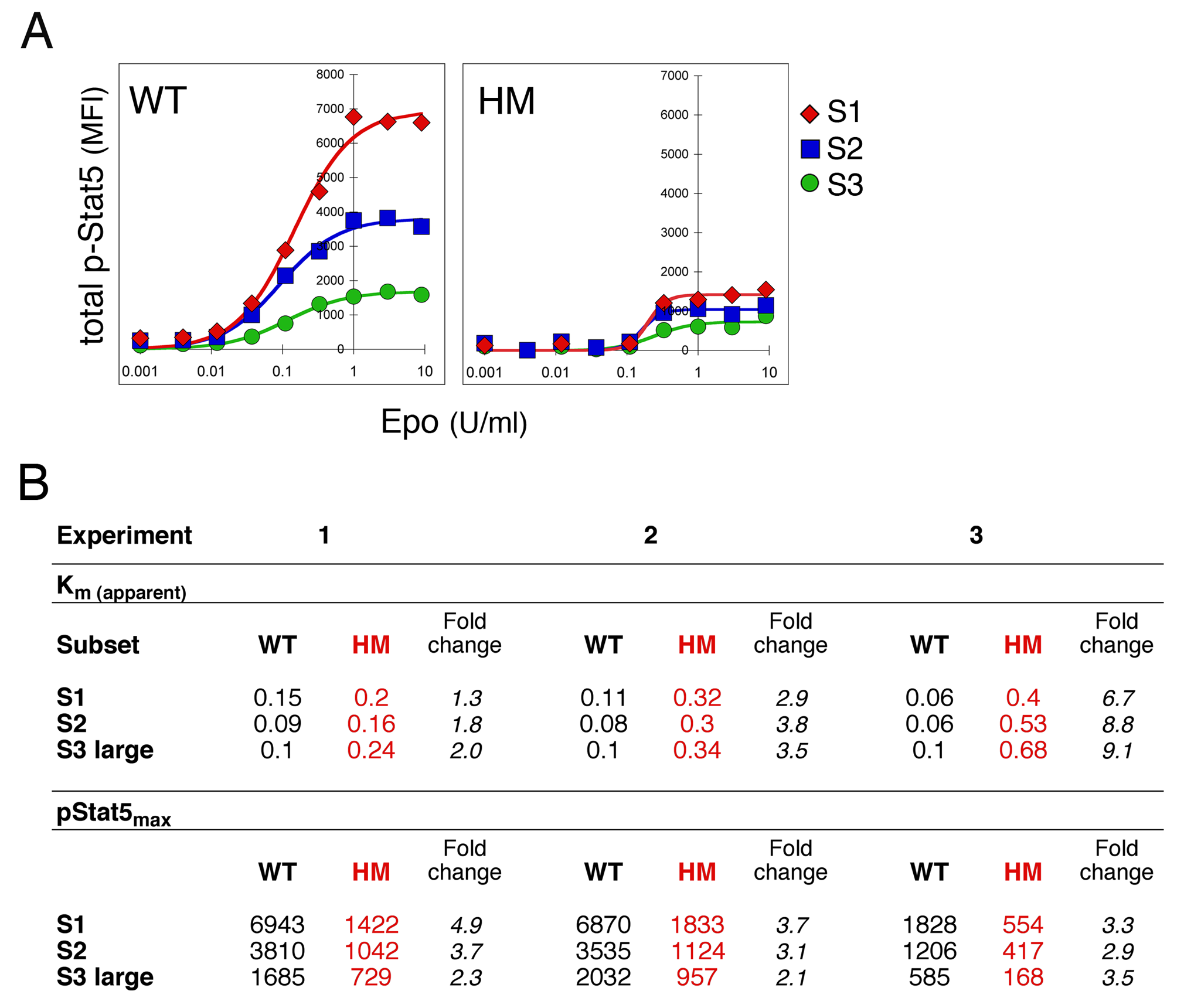

Supplement: Figure S3 — Binary p-Sta5 signaling in EpoR-HM erythroblasts. (A) Representative plots of “total p-Stat5 versus Epo concentration” for subsets S1 to S3 in wild-type (left panel) and EpoR-HM (right panel) fetal liver. The same experiment as in Figure 3C, main text. (B) Three independent experiments assessing p-Stat5 signaling in EpoR-HM mice. Values for p-Stat5max and apparent Km were obtained by fitting Hill curves to plots of “total p-Stat5 MFI versus Epo concentration” of the type illustrated in (A). The Hill equation was used as follows: , where S = Epo concentration in U/ml, and p-Stat5 is the total p-Stat5 fluorescence; best fit was obtained by varying n ( = Hill coefficient, “nH” in the text), Km ( = the apparent Km), and p-Stat5max ( = the maximal p-Stat5 response to high Epo), using the solver function of Microsoft Excel. R 2 is Pearson's product moment correlation coefficient, correlating experimental data with values predicted by the Hill equation for the corresponding Epo concentrations. The Hill coefficients and R 2 values for this analysis are shown in Figure 3E, main text. The Km and p-Stat5max were all significantly different in wild-type and EpoR-HM mice (paired t test, p = 0.003 and 0.023, respectively). (TIF) [file pbio.1001383.s003.tif]

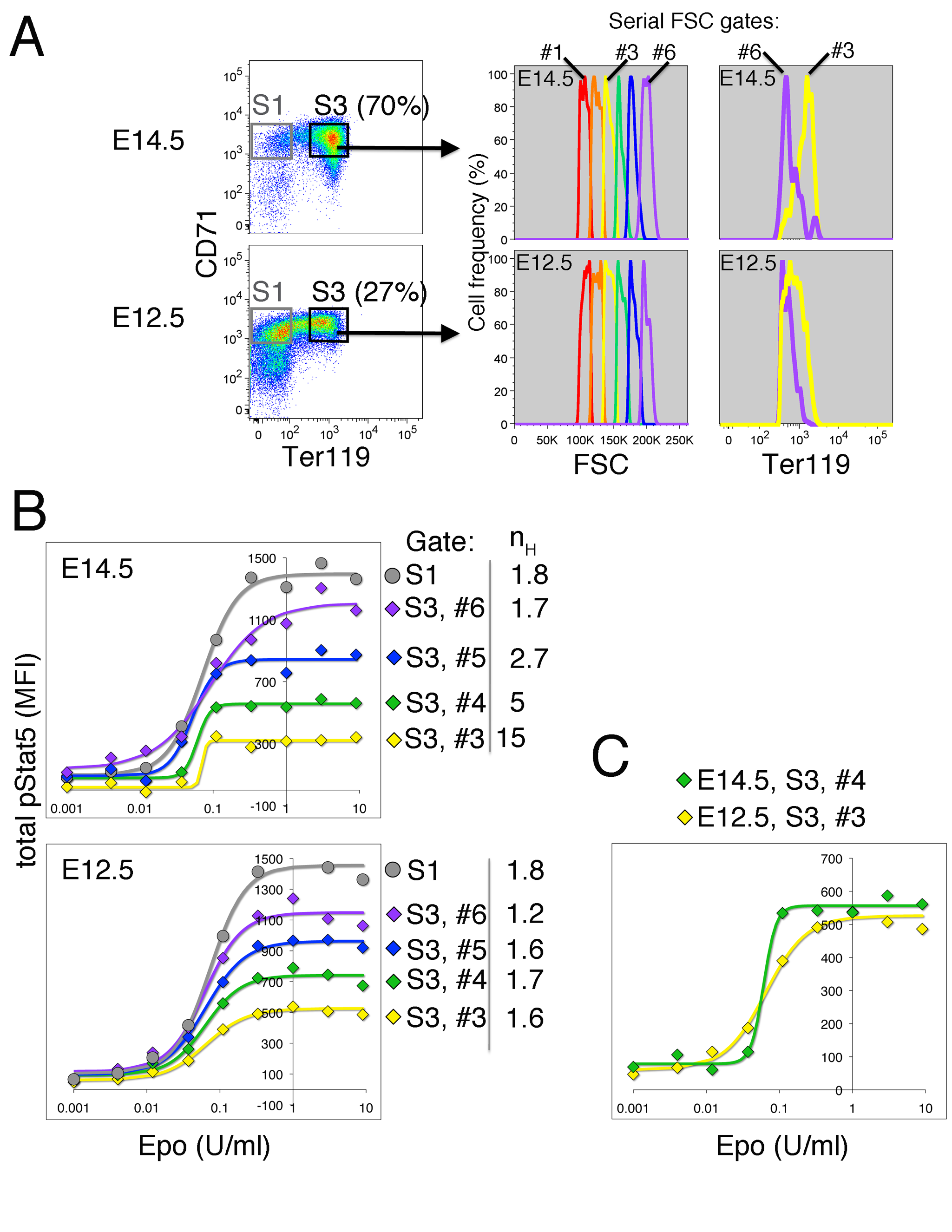

Supplement: Figure S4 — Comparison of dose/response curves in E12.5 and E14.5 embryos. E12.5 and E14.5 fetal livers were stimulated with Epo and analyzed by flow cytometry in the same experiment. The data for the E14.5 fetal liver are also shown in Figure 3F–G. (A) CD71/Ter119 profiles of E12.5 and E14.5 fetal livers (left panels). The S3 subsets were divided into serial FSC gates, each corresponding to 300 channels (middle panels). Right panels show an overlay of Ter119 expression in FSC gates #3 and #6 for each of the embryos. (B) Epo dose/p-Stat5 response curves for the S3 FSC gates in the E12.5 and E14.5 embryos. Gate number and corresponding Hill coefficient are shown for each curve. (C) Overlay of the indicated dose/response curves. (TIF) [file pbio.1001383.s004.tif]

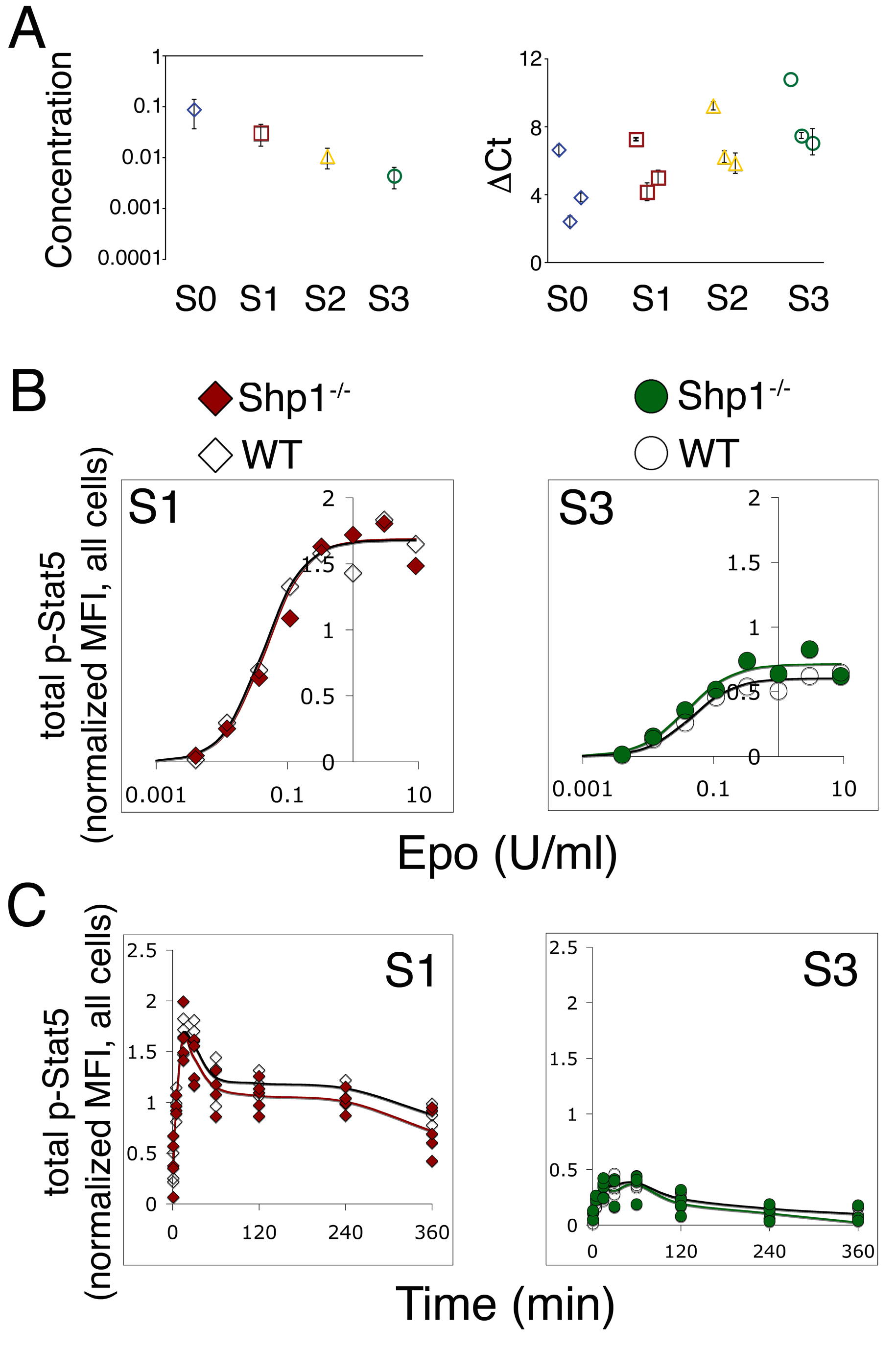

Supplement: Figure S5 — Stat5 signaling in Shp1−/− (C57BL/6J-Ptpn6me/J) fetal liver. (A) Quantitative real-time PCR for Shp1 in sorted S0–S3 subsets from wild-type embryos (E12.5–E13.5). The left panel shows Shp1 mRNA relative to β actin (mean ± SE), calculated from three independent experiments. The right panel shows the ΔCt ± SE for each individual experiment. (B) Epo dose/p-Stat5 response analysis in Shp1−/− fetal liver fitted with Hill curves. Data (mean) from two independent experiments, each with one embryo of each genotype. Normalization as in Figure 1E. (C) Time course of p-Stat5 response to Epo stimulation with 0.2 U/ml, in Shp1−/− embryos (n = 5) and in matched controls (n = 3). Data points are measurements in individual embryos. (TIF) [file pbio.1001383.s005.tif]

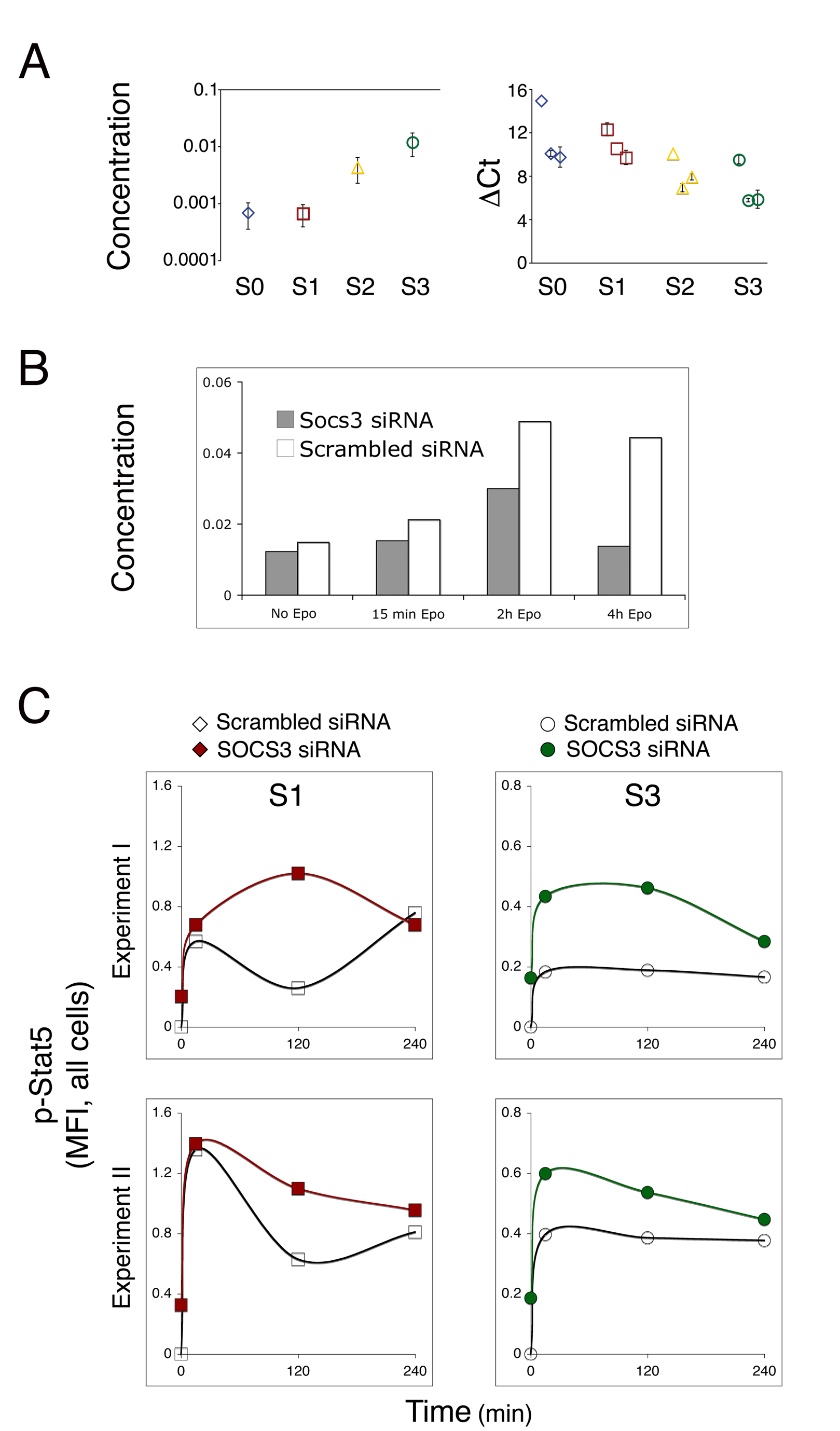

Supplement: Figure S6 — SOCS3 regulation of Stat5 signaling. (A) Quantitative real-time PCR for SOCS3 in sorted S0–S3 subsets from wild-type embryos (E13–E14.5). The left panel shows SOCS3 mRNA relative to β actin (mean ± SE), calculated from three independent experiments. The right panel shows the ΔCt ± SE for each individual experiment. (B, C) Fetal liver cells were electroporated with SOCS3 siRNA or with “scrambled” control siRNA. Four hours later cells were stimulated with Epo. Both SOCS3 mRNA (B) and the p-Stat5 response (C) were measured at the indicated times. (TIF) [file pbio.1001383.s006.tif]

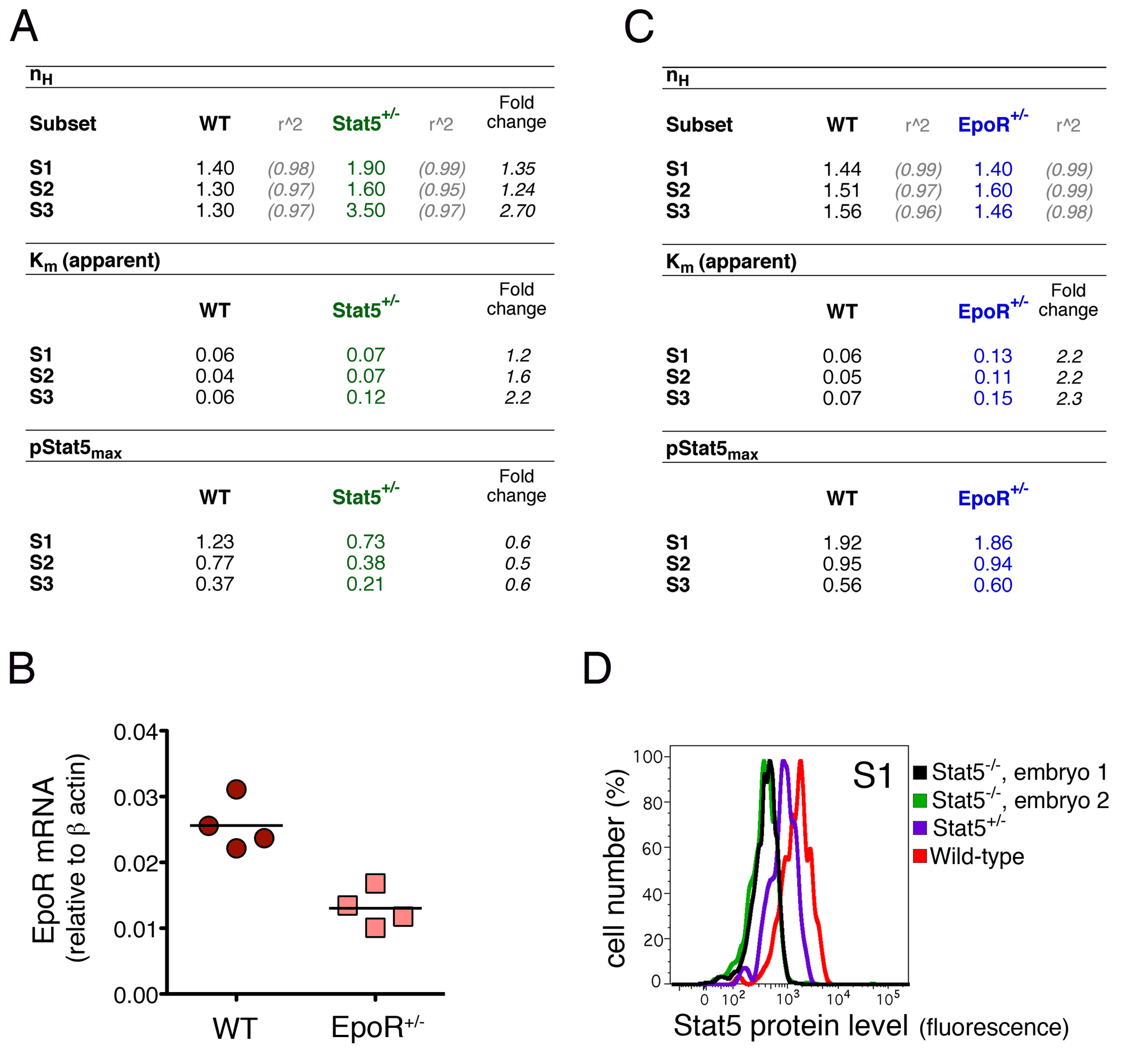

Supplement: Figure S7 — p-Stat5 signaling in Stat5+/− and EpoR+/− erythroblasts. (A) Epo Dose/p-Stat5 response analysis of Stat5+/− fetal liver. Values for the Hill coefficients, p-Stat5max, and apparent Km were obtained by fitting Hill curves (see legend to Figure S3B) to the “total p-Stat5 MFI versus Epo concentration” data (main text, Figure 4B). Note a substantially lower p-Stat5max in Stat5+/− embryos. A total of seven Stat5+/− and six control embryos were individually analyzed. R 2 is Pearson's product moment correlation coefficient. (B) EpoR mRNA in wild-type (WT, circles) and EpoR+/− (squares) fetal liver, measured using quantitative RT-PCR at E13.5. Data points represent measurements in individual embryos, and are expressed relative to β actin mRNA. Mean values for each genotype are denoted with a black line. (C) Epo Dose/p-Stat5 response analysis of EpoR+/− fetal liver. Analysis as for Stat5+/− embryos in panel A, of data presented in Figure 4C. Note doubling of the apparent Km in EpoR+/− embryos (resulting in a shift of the curve to the right). A total of four EpoR+/− and three control embryos were analyzed independently. (D) Measurements of Stat5 protein levels in fetal liver subsets using flow-cytometry. Fixed and permeabilized fetal liver cells were labeled with antibodies against Ter119 and CD71, and in addition, with a rabbit polyclonal antibody, which recognizes Stat5 regardless of its state of activation (ab 7969, Abcam) and a secondary anti-rabbit IgG antibody conjugated to APC. Flow cytometry histograms reflecting total Stat5 protein expression (as APC fluorescence) are shown for two Stat5−/− embryos (these provide the non-specific background fluorescence), and for one wild-type and one Stat5+/− embryo. Stat5 expression in Figure 4D–F was determined as Stat5 MFI, with background fluorescence subtracted, and expressed as a ratio to the average Stat5 MFI of S1 cells. (TIF) [file pbio.1001383.s007.tif]

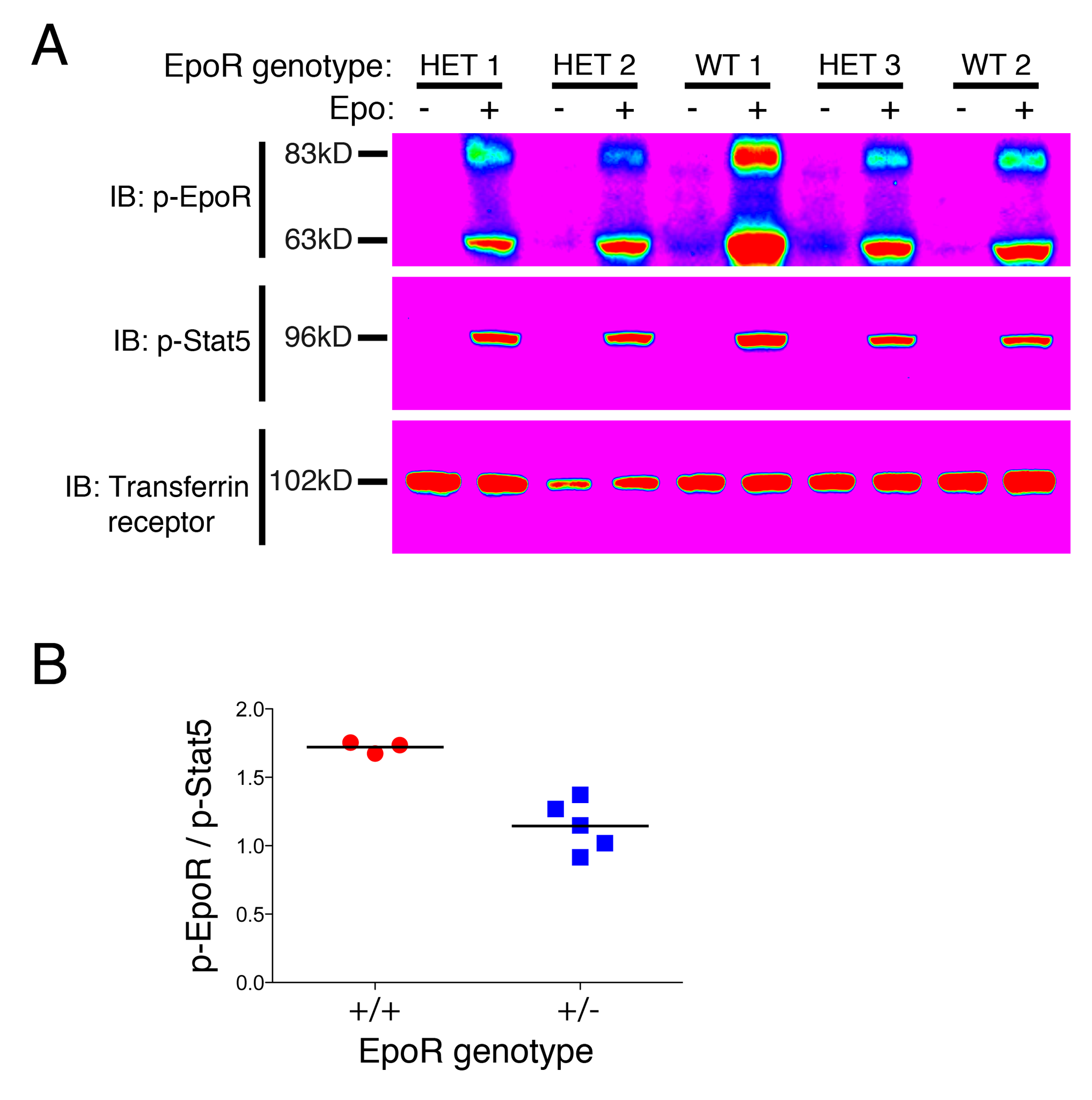

Supplement: Figure S8 — EpoR expression is not limiting to the maximal p-Stat5 signal. Ter119-negative cells were sorted from E13.5 fetal livers using magnetic beads. The cells from each fetal liver were divided into two aliquots, one of which was stimulated with Epo. The entire lysate from each aliquot was resolved using SDS-PAGE and analyzed by Western blotting. Two independent litters were processed. (A) Western blot analysis of embryos from a single litter. The membrane was stripped and re-probed sequentially with antibodies to p-EpoR, p-Stat5, and the transferrin receptor. The transferrin receptor signal indicates the presence of S1 erythroblasts. Chemiluminescence was quantitated using Bio-Rad Molecular Imager Chemi Doc XRS+ and Image Lab Software Version 3.0.1, Bio-Rad Laboratories. The pseudo-color is a software tool indicating increasing signal intensity, in the order pink ( = background), blue, green, and red ( = maximal). All signals were within the linear range as confirmed by sequential automated timed exposures. (B) The p-EpoR/p-Stat5 ratio for wild-type and EpoR+/− embryos. Each data point is derived from a single fetal liver. Three wild-type and five EpoR+/− fetal livers were pooled from two litters. Ratios were 1.7±0.02 for wild-type, 1.1±0.08 for EpoR+/−, p = 0.002, two-tailed t test with unequal variance. (TIF) [file pbio.1001383.s008.tif]

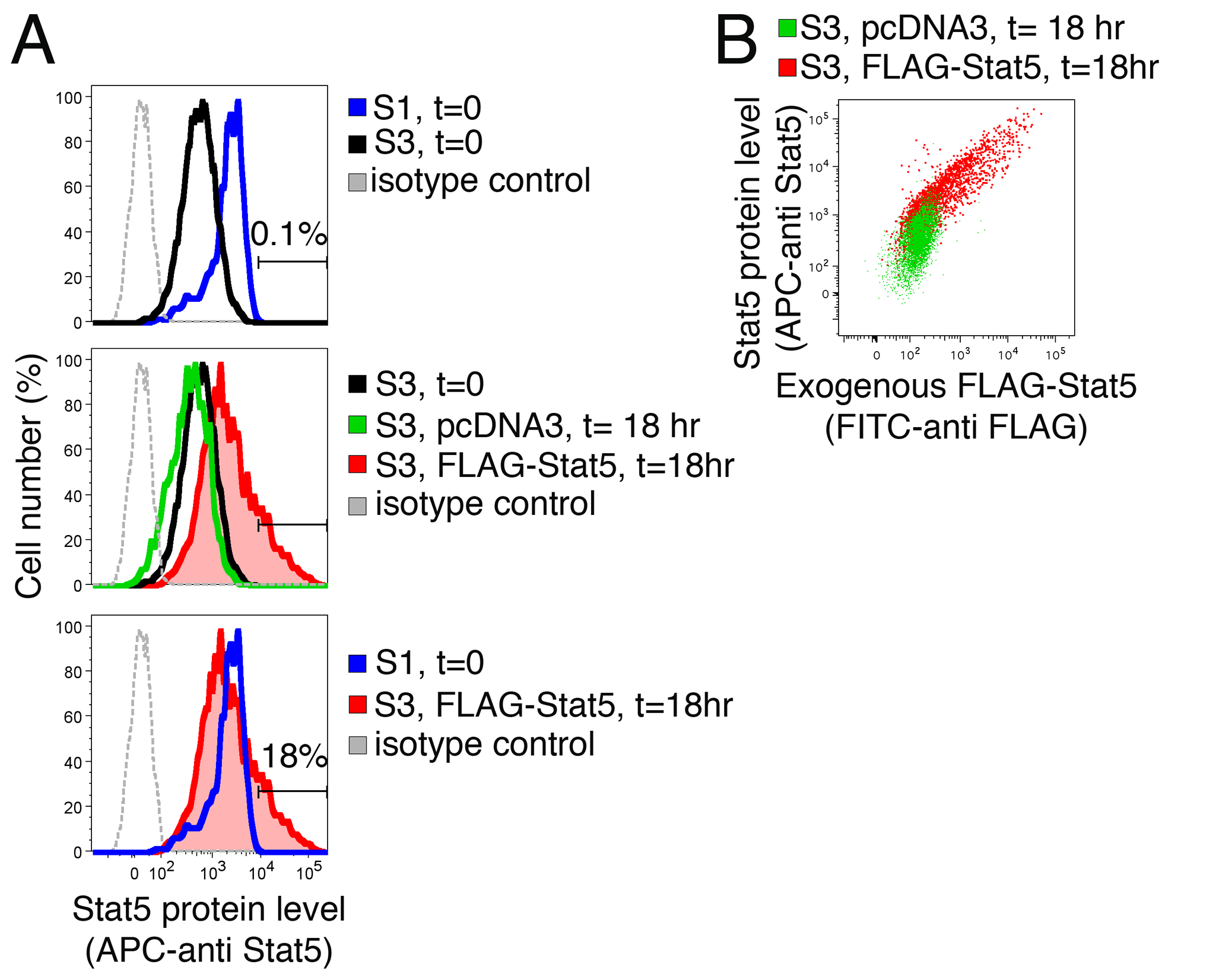

Supplement: Figure S9 — Measurement of exogenous Stat5 protein in transfected fetal liver cells. (A, B) Fetal liver cells were electroporated with FLAG-tagged Stat5a (“FLAG-Stat5”) or with control “empty vector” (“pcDNA3”). Cells were incubated overnight in the presence of Epo (0.2 U/ml), to allow expression of the transduced constructs. Expression of exogenous FLAG-Stat5 protein and p-Stat5 signaling were measured at 18 h. (A) Stat5 protein levels are assessed as in Figure S7D. Top panel, Stat5 protein in freshly isolated S1 and S3 cells, prior to transfection with FLAG-Stat5. Middle panel, Stat5 protein in S3 cells transduced with either FLAG-Stat5 (red) or with empty vector (pcDNA3, green). Stat5 protein in fresh S3 cells (black) is shown for comparison. Lower panel, Stat5 protein in S3 cells transduced with FLAG-Stat5 (red), compared with Stat5 protein levels in fresh S1 cells (blue). (B) Measurement of FLAG fluorescence is an accurate assessment of exogenous Stat5 protein levels. S3 cells transfected with either FLAG-Stat5 (red) or empty vector (green), labeled with both anti-FLAG antibody (x-axis) and an anti-Stat5 antibody (y-axis). A linear correlation is observed between FLAG and Stat5 staining in FLAG-Stat5 transfected cells. (TIF) [file pbio.1001383.s009.tif]
