# Supplementary material for: Stat5 Signaling Specifies Basal versus Stress Erythropoietic Responses through Distinct Binary and Graded Dynamic Modalities
Source: PLoS Biol. 2012 Aug 28;10(8):e1001383. doi: 10.1371/journal.pbio.1001383 (PMC3433736; doi:10.1371/journal.pbio.1001383)
Supplement: Text S1 — Supplementary analysis: Cellular Stat5 is limiting during Stat5 phosphorylation in erythroid cells. (PDF) [file pbio.1001383.s010.pdf]

Supplementary Analysis:

Cellular Stat5 is limiting during Stat5 phosphorylation in erythroid cells

Our data shows that maximal p-Stat5 produced in response to Epo dosing (='p-Stat5_{max}') is linearly related to total cellular Stat5 (Figures 4F, 5D). This linear relationship is maintained across all erythroid progenitor stages and over a wide range of cellular Stat5 expression levels, whether endogenous, or exogenously introduced into cells. By contrast, p-Stat5_{max} is not affected by the reduced expression of EpoR in $\text{EpoR}^{+/-}$ cells (Figures 4C, S7B, S8). It is also apparently unaffected by the decrease in cell-surface EpoRs known to occur with progenitor differentiation [1], since introduction of exogenous Stat5 into late erythroid progenitors increases p-Stat5_{max} linearly with cellular Stat5 (Fig 4F).

These findings suggest that the limiting parameter for p-Stat5_{max} is cellular Stat5, rather than the number of cell-surface EpoRs. Therefore, conditions of substrate excess, required for Michaelis-Menten kinetics, do not apply to Stat5 phosphorylation in erythroid cells. Instead, the analysis below shows that our results are consistent with conditions of enzyme excess. Under such conditions, maximal product formation is linearly related to initial substrate concentration [2] [3].

Analysis:

Epo binds a pre-formed complex of EpoR and Jak2, to form an active enzyme complex, or 'E':

$$\text{Epo} + (\text{EpoR-Jak2}) \rightleftharpoons (\text{Epo-EpoR-Jak2}) \quad (1)$$

and

$$E = (\text{Epo-EpoR-Jak2}) \quad (2)$$

The activated enzyme complex 'E' binds Stat5, to form an enzyme-substrate complex 'C', which phosphorylates Stat5:

$$E + \text{Stat5} \underset{k_{-1}}{\overset{k_1}{\rightleftharpoons}} C \xrightarrow{k_2} \text{p-Stat5} + E \qquad (3)$$

Let initial Stat5 concentration be denoted as 'S_0' and the concentration of the product, p-Stat5, as 'P'. From reaction (3), the rate of p-Stat5 formation, dP/dt, is provided by

$$\frac{dP}{dt} = k_2 C \qquad (4)$$

If the active enzyme concentration is in great excess of 'S_0', that is, $E \gg S_0$, then reaction (1) is essentially independent of reaction (3). Namely, the binding of Stat5 to E will not appreciably decrease E from its value as set by reaction:

$$E \approx E_0 \qquad (5)$$

where E_0 is the initial active enzyme concentration[1]. More generally, this assumption holds whenever the fraction of substrate-bound enzyme is negligible: $\frac{C}{E} \ll 1$; such conditions arise not only at pure enzyme excess, $E \gg S_0$, but also under the weaker requirement that, $E + K_m \gg S_0$, where $K_m = \frac{k_{-1} + k_2}{k_1}$ is the Michaelis-Menten constant of reaction (3) [3].

[1] Note that strictly speaking E_0 is not the initial active enzyme concentration, but the equilibrium concentration implied by reaction (1).

Under these conditions,

$$C = \frac{E_0(S_0 - P)}{K_m + E_0} \tag{6}$$

From (4), (5) and (6), the rate of formation of p-Stat5 $(=P)$ is given by

$$\frac{dP}{dt} = k_2 \frac{E_0(S_0 - P)}{K_m + E_0} \tag{7}$$

If we let $K = \frac{k_2 E_0}{K_m + E_0}$, equation (7) now becomes

$$\frac{dP}{dt} = K(S_0 - P)\,. \tag{8}$$

From (8), and the initial condition $P = 0$, the concentration of the product, p-Stat5 increases with time as

$$P = S_0(1 - e^{-Kt}) \tag{9}$$

Equation (9) is in agreement with our observations in Figures 4F and 5D (main text), that the maximal value for *P*, 'p-Stat5$_{max}$' is independent of E_0 and is a linear function of the initial Stat5 substrate concentration, S_0. Therefore, our observations are consistent with the initial assumption of this analysis, namely that the active enzyme complex is in excess of cellular Stat5 concentration.

Supplementary References:

1. Zhang J, Socolovsky M, Gross AW, Lodish HF (2003) Role of Ras signaling in erythroid differentiation of mouse fetal liver cells: functional analysis by a flow cytometry-based novel culture system. Blood 102: 3938-3946.
2. Tzafriri AR (2003) Michaelis-Menten kinetics at high enzyme concentrations. Bull Math Biol 65: 1111-1129.
3. Tzafriri AR, Edelman ER (2007) Quasi-steady-state kinetics at enzyme and substrate concentrations in excess of the Michaelis-Menten constant. J Theor Biol 245: 737-748.
