# Supplementary material for: Stat5 Signaling Specifies Basal versus Stress Erythropoietic Responses through Distinct Binary and Graded Dynamic Modalities
Source: PLoS Biol. 2012 Aug 28;10(8):e1001383. doi: 10.1371/journal.pbio.1001383 (PMC3433736; doi:10.1371/journal.pbio.1001383)
Supplement: Text S2 — Supplementary materials and methods. (PDF) [file pbio.1001383.s011.pdf]

Supplementary Materials and Methods

Mice

BALB/c ByJ, C57BL.6J and C57BL/6J-Ptpn6^{me}/J strains were purchased from the Jackson Laboratory (Maine). Stat5-null were supplied by Dr. Lothar Hennighausen, NIDDK, Bethesda, MD, and EpoR-H and EpoR-HM by Dr Jim Ihle, St. Jude Children's Research hospital, Memphis, TN. Mice were matched for the same background strain and embryonic age in all experiments.

DNA constructs and cloning

pcDNA3-Flag Stat5a, pcDNA3-Flag Stat5 1*6 were generated by cloning the Flag-Stat5 (wild type or mutant), provided by Dr. T. Kitamura, in the pcDNA3 construct (Invitrogen). Retroviral constructs were previously described (Socolovsky et al, 1999, Cell, 98: 181-91). pcDNA3-Flag Stat5a-Y694F was generated by mutagenesis using the QuickChange Site-Directed Mutagenesis Kit (Stratagene) according to the manufacturer's instructions.

Quantitative RT-PCR

RNA was extracted, using the AllPrep DNA/RNA Micro Kit (Qiagen), from FACS-sorted fetal liver cells. Taqman real time PCR (Applied Biosystems) EpoR was performed on cDNA prepared using the SuperScript First-Strand kit (Invitrogen). The Mm 01175895 g1 probe (Applied Biosystems) was used for EpoR expression analysis.

SOCS3 siRNA

Fetal liver cells were electroporated with 2μg of SOCS3 (L-040626-01, Dharmacon) or control (D-001810-10, Dharmacon) siRNA. Cells were recovered in 20% serum for 4 hours and subsequently stimulated with Epo (0.2 U/mL) in a time course experiment.

Quantitative western blot analysis for phospho-EpoR (p-EpoR) and p-Stat5

E13.5 embryos from EpoR$^{+/-}$ x EpoR$^{+/+}$ matings were stimulated with 2U/ml Epo for 5 minutes and lysed immediately in an NP40 lysis buffer. Embryos from 2 litters were analyzed by western blotting in parallel (each litter on a separate membrane). Membranes was stripped and re-probed sequentially with antibodies to p-EpoR (1:200, Santa Cruz Biotechnology, Inc. sc-20236-R), p-Stat5 (1:1000, BD, 611964) and transferrin (1:1000, Invitrogen,136800). The signal from the membranes was obtained using Bio-Rad Molecular Imager Chemi Doc XRS+**.** Western blots were quantified using Image Lab Software Version 3.0.1, Bio-Rad Laboratories, using automated sequential timed exposures. We ascertained that all signals were within the linear range of the assay. Each reading was the mean of at least 3 timed exposures (normalized to the same band for all exposures).
